# Transcriptomic changes in single yeast cells under various stress conditions

**DOI:** 10.1186/s12864-023-09184-w

**Published:** 2023-02-24

**Authors:** Yangqi Su, Chen Xu, Jonathan Shea, Darla DeStephanis, Zhengchang Su

**Affiliations:** Department of Bioinformatics and Genomics, The University of North Carolina at Charlotte, 28223 Charlotte, NC USA

**Keywords:** Single cell, RNA-seq, Yeast, Stress response, Gene regulatory networks

## Abstract

**Background:**

The stress response of *Saccharomyces cerevisiae* has been extensively studied in the past decade. However, with the advent of recent technology in single-cell transcriptome profiling, there is a new opportunity to expand and further understanding of the yeast stress response with greater resolution on a system level. To understand transcriptomic changes in baker’s yeast *S. cerevisiae* cells under stress conditions, we sequenced 117 yeast cells under three stress treatments (hypotonic condition, glucose starvation and amino acid starvation) using a full-length single-cell RNA-Seq method.

**Results:**

We found that though single cells from the same treatment showed varying degrees of uniformity, technical noise and batch effects can confound results significantly. However, upon careful selection of samples to reduce technical artifacts and account for batch-effects, we were able to capture distinct transcriptomic signatures for different stress conditions as well as putative regulatory relationships between transcription factors and target genes.

**Conclusion:**

Our results show that a full-length single-cell based transcriptomic analysis of the yeast may help paint a clearer picture of how the model organism responds to stress than do bulk cell population-based methods.

**Supplementary Information:**

The online version contains supplementary material available at 10.1186/s12864-023-09184-w.

## Background

The ability to adapt to a changing environment is crucial to survival of individual cells, and this is even more evident for single celled organisms [1–3]. A rapid change in a cell’s surrounding induces stress, which requires the cell to activate complex mechanisms of sensing and signal transduction to adapt [4–6]. Such mechanisms eventually lead to the expression of genes and proteins, which can often be specific to the type of stress encountered. At the same time, organisms also develop general responses regardless of the type of stress encountered to better deal with a constantly changing environment [7]. The study of stress response in various organisms has led to a better understanding of some of the fundamental aspects of cellular biology [2, 3, 5, 8].

One such model organism that has been studied extensively is the budding yeast *Saccharomyces cerevisiae*. The budding yeast is a single-cell organism that faces a constantly changing environment when living freely in nature, often having to deal with multiple types of stress at the same time. For many decades, the responses of budding yeast to different environmental perturbations have been studied systematically on different levels, leading to the discovery and understanding of many pathways of the organism [1, 2, 9, 10]. On the other hand, while it may be the most well understood eukaryotic organism, we are still far from being able to completely model this organism’s response to stress. The advent of microarray technology meant simultaneous profiling of thousands of genes of yeast was possible [11]. These developments led to the early analysis of the transcriptomic responses of yeast to different environmental changes, resulting in the discovery of a set of roughly 900 genes, termed the yeast environmental stress response (ESR) genes, which were activated as a general response to multiple types of stress [2]. Up-regulated ESR genes were found to be regulated by transcription factors (TFs) Msn2p and Msn4p and related directly to mitigation of stress, while down-regulated ESR genes were found to be involved in ribosomal biogenesis and protein synthesis [1, 12, 13]. This general response was thought to be crucial for the survival of yeast cells in preparation for changes in the environment [2].

The yeast response to amino-acid starvation has been shown to be mediated through the general amino acid control (GAAC) pathway [14], and more specifically through the TF Gcn4p and protein kinase Gcn2p. Gcn2p phosphorylates translation initiation factor Eif2p, thus inhibiting overall translation rates [15]. Though the abundance of Gcn4p is controlled at the translational level, phosphorylation of Eif2p increases the level of Gcn4p via a mechanism involving delayed ribosomal re-initiation and inhibitory upstream ORFs in the 5’ region of the *GCN4* gene ([16], thereby Gcn4p activates the promoters of genes that harbor a GCN response element [17]. Early transcriptomic studies showed that amino-acid starvation down-regulated genes related to growth and ribosome biogenesis, while upregulating genes involved in amino-acid biosynthesis, cellular redox reaction, carbohydrate metabolism, cell wall modification, protein folding and degradation, DNA damage repair, fatty acid metabolism, metabolite transport, and autophag [18].

The yeast has also been studied extensively for responses to changes in carbon sources [16–19]. Though yeast prefers glucose as a carbon source during fermentative growth, it also can utilize other carbon sources as alternatives [19]. During growth in glucose-rich cultures, genes in pathways for utilizing alternative carbon sources, such as galactose, maltose and sucrose, are repressed through a glucose sensitive repressor Mig1 [20]. It was found that glucose starvation upregulated genes were involved in oxidative phosphorylation and the TCA cycle, and some of them encode high-affinity glucose transporters [21]. At the same time, glucose starvation results in a drastic reduction in transcription rates and degradation of mRNA as well as almost complete inhibition of translational machinery [22, 23]. Another study profiling ribosome of yeast under glucose starvation noted that while overall protein synthesis was reduced, transcription of many stress-response and glucose-repressed genes was increased [20, 22].

As a single-celled organism, yeast in nature may constantly experience sudden changes in surrounding osmolarity. The adaptation of yeast to hyperosmotic stress has been studied extensively, whereby a sudden increase in osmolarity will cause the yeast cell to shrink and the high osmolarity glycerol response pathway is activated [24]. Less is known about an osmolarity downshift, when there is a rapid influx of water, leading to increase of cell size and turgor pressure, during which the cell wall plays a vital role in preventing the cell from bursting [25]. This process initiates the cell integrity pathway (CWI). Glycerol export is mediated via the Fps1p transporter. An influx of calcium ions also occurs [26], which results in the activation of the TF Crz1p. The osmolarity sensor Sln1p, the phosphotransferase Ypd1p, and the TF Skn7p form a phosphor-relay system that activates Skn7p by phosphorylation upon cell swelling [27, 28]. Upon activation, Skn7p activates the transcription of genes related to cell wall biogenesis [27, 28]. Micro-array transcriptomic analysis revealed a reversal of the gene expression response during hyper-osmolarity stress but failed to find a distinct pattern of gene expression during hypotonic stress. [2]

In the past decade, the rapid development of high throughput RNA-seq technology has provided a better platform than microarray to study the transcriptomic profiles of organisms, providing the ability to quantify gene expression genome-wide in a single assay with higher resolution, larger dynamic ranges and lower technical [29–31]. Moreover, the advent of single-cell RNA-seq (scRNA-seq) methods has provided an unprecedented opportunity to study the response of individual cells of an [32]. This is even more evident in the case for the budding yeast, which is itself a single celled organism. Utilizing scRNA-seq, it may now be possible to delve deeper into the intricacies of an individual yeast cell’s response to different stress. However, studies applying single-cell transcriptomic studies in micro-organisms such as the budding yeast have been limited in comparison to studies of larger mammalian cells. Recent microfluidics-based methods have allowed high throughput scRNA-seq studies to be [33]. Most notably, methods such as those developed by 10x Genomics have allowed the simultaneous sequencing of hundreds of thousands of cells [34]. Though these methods are well optimized for mammalian cells, they are not easily applicable to yeast cells, due to the complexity introduced in sample preparation by having to lyse individual microbial cell walls, as well as the relatively low amounts of mRNA in small microbial cells compared to mammalian cells. Consequently, though studies of yeast stress responses have been extensively carried out throughout the past decade using a variety of techniques, scRNA-seq based studies have been relatively few.

To fill this gap, we adapted a full-length scRNA-seq method to profile transcriptomes of single yeast cells under hypotonic osmolarity, glucose starvation or amino acid starvation. Although our sample size is not on the scale of droplet-based scRNA-seq methods that only sequence the 3’-end of mRNA molecules and are more prone to drop-out effects due to shallow sequencing depths, we sequenced each single-cell transcriptome in full-length to a sufficient depth and are thus less prone to drop-out effects. Furthermore, a single-cell approach allows for more biological repeats than a bulk-based procedure. Using these data, we not only confirmed many earlier findings based on bulk cell data, but also reveal novel aspects of stress responses in yeast at single-cell level.

## Results

### Transcriptomes of single yeast cells are sufficiently sequenced

Using the scRNA-seq protocol, we sequenced the full-length transcriptomes of 117 yeast cells from four treatments in two sequencing batches: amino acids starvation (AAS) (n = 20 in batch 1), isotonic (n = 19 in batch 1; n = 19 in batch 2), glucose starvation (GS) (n = 47 in batch 2) and hypotonic conditions (n = 12 in batch 1). As summarized in **Table S1** in **Supplementary File 1**, an average of 9.5 million reads were generated in each cell, and an average of 36% and 11% of them were uniquely mapped to the genome and ERCC spike-in RNA sequences, respectively. Moreover, we sequenced a total of 59 RNA-seq libraries prepared using varying amounts (5pg, 10pg, 20pg, 100pg, 1,000pg and 10,000pg) of bulk RNA from yeast cells under AAS using the same protocol. As summarized in **Table S2** in **Supplementary File 1**, an average of 6.7 million reads were generated in bulk samples, and an average of 34% and 24% of them were uniquely mapped the genome and ERCC spike-in RNA sequences, respectively.

**Fig. 1:**
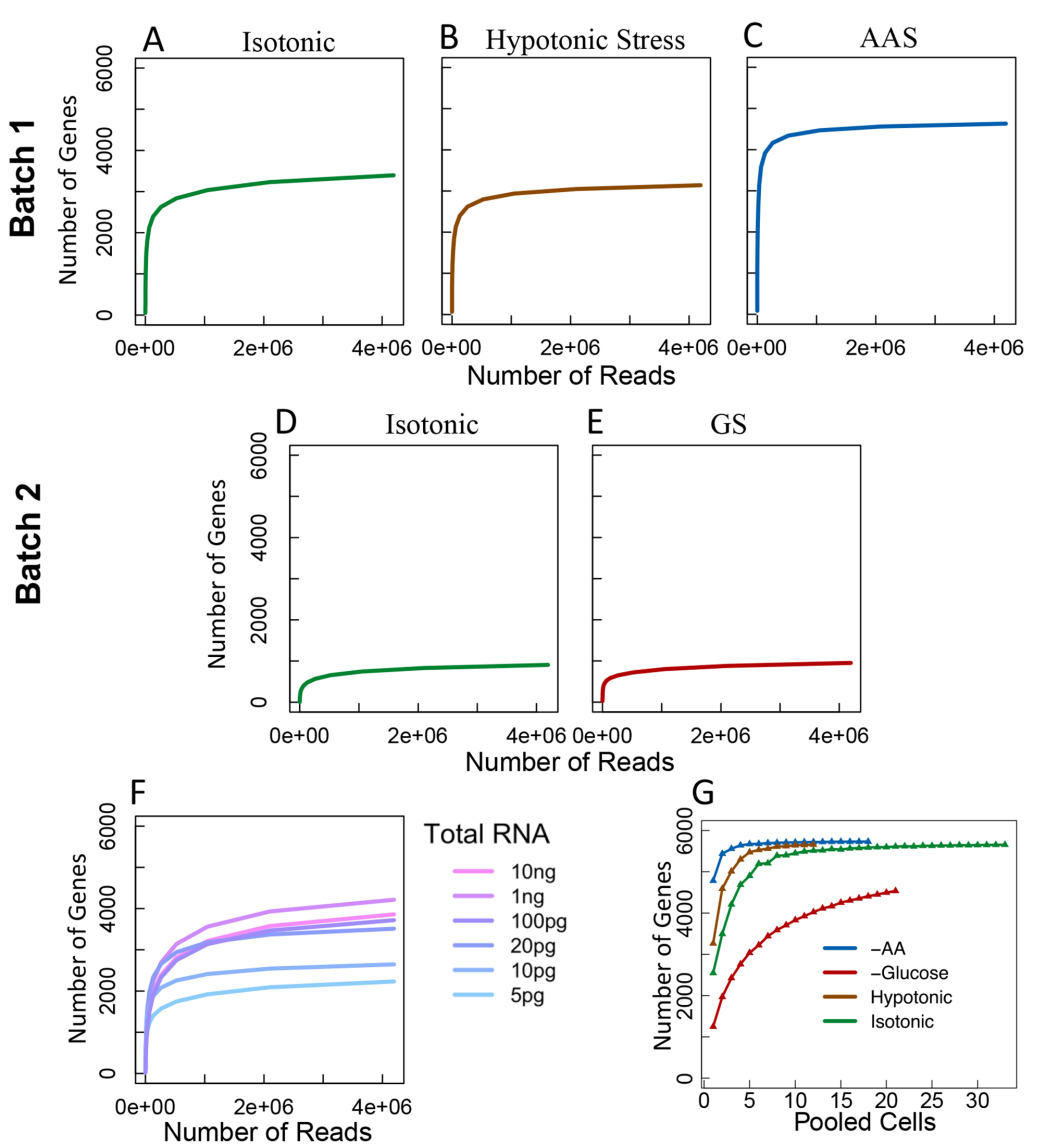
Saturation of detected genes in the libraries. A. Number of genes detected in each cell under isotonic condition from batch 1 as a function of number of mapped reads randomly sampled from the cell. B. Number of genes detected in each cell under hypotonic stress samples from batch 1 as a function of number of mapped reads randomly sampled from the cell. C. Number of genes detected in each cell under AAS from batch 1 as a function of number of mapped reads randomly sampled from the cell. D. Number of genes detected in each cell under GS from batch 2 as a function of number of mapped reads randomly sampled from the cell. E. Number of genes detected in each cell under isotonic condition from batch 2 as a function of number of mapped reads randomly sampled from the cell. F. Number of genes detected in each bulk sample as a function of number of mapped reads randomly sampled from the sample. The bulk libraries were prepared using different amount (5pg, 10pg, 20pg, 1,00pg, 1000pg and 10,000pg) of input mRNA extracted from a population of cells under AAS. G. Number of genes detected as a function of number of single cells randomly selected from those under the same conditions. Bold colored lines represent average detections

To see whether the sequencing depth was sufficient to cover all the captured mRNAs in a cell, we randomly sampled different numbers of mapped reads from the scRNA-seq libraries and computed the total number of genes to which reads were mapped. As shown in Fig. 1E, the number of genes detected approached saturation when around 1 million reads were sampled for each cell, suggesting that for most of our scRNA-seq libraries, the sequencing depth should be more than sufficient to detect most transcribed mRNAs. However, there is considerable variation in the number of genes detected between cells under the same treatment (Fig. 1A ~ 1E). Comparing the two different batches of isotonic treatment (Fig. 1A and E) reveals that batch effect contributes significantly to the difference in the number of genes detected, a well-known phenomenon for scRNA-seq libraries [35–38]. On the other hand, the more input bulk mRNA amount, the more genes detected in the bulk mRNA samples (Fig. 1F). Therefore, the varying numbers of genes detected in single cells are most likely caused by varying amounts of mRNA that each cell expressed or released during lysis, which is characteristic of scRNA-seq libraries [39], thus partially explaining the variation in detection rates of genes. Bootstrapping single cells by computationally pooling raw reads from a set of single cells under the same treatment shows, except for GS, the detection rate already reaches saturation when reads from as few as five cells were combined (Fig. 1G), indicating that aggregating over the single cells can provide a comparable sample to that of bulk analysis.

**Fig. 2:**
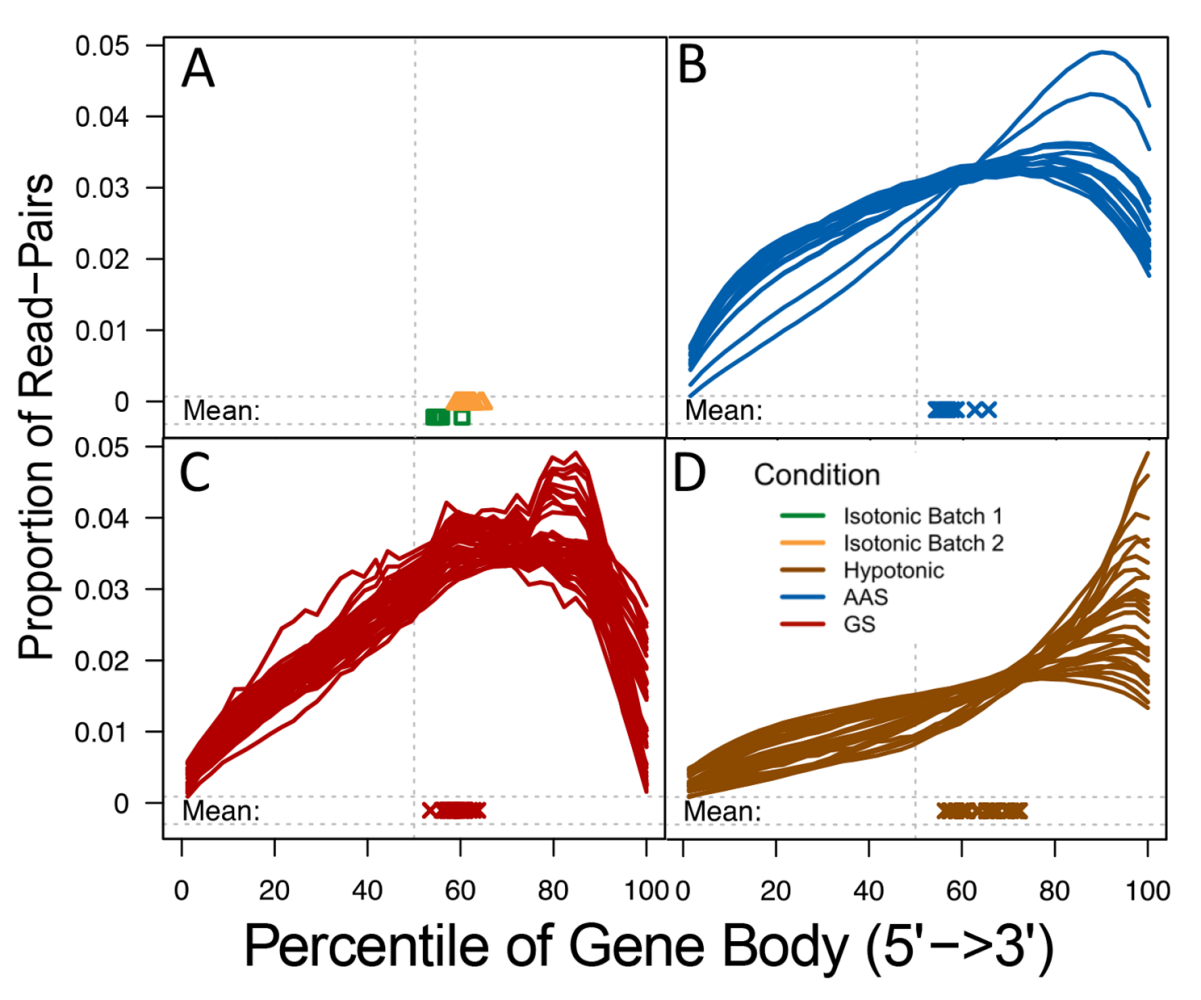
Reads coverage along the 5’-end to the 3’-end of the coding regions of genes. For each library, the averaged relative coverage is shown at each relative position along the length of coding regions of genes from the 5’-end to the 3’-end. **A**. Isotonic condition, batch 2 samples are colored orange here to distinguish from batch 1 samples (colored green). **(B)** AAS. **(C)** GS. **(D)** Hypotonic stress

An evaluation of the possible bias of read coverage along a gene body for all libraries show that the read coverage declines toward the 5’-end for all libraries (Fig. 2). This result is consistent with the earlier findings that single-cell RNA-Seq data tend to be biased toward the 3’ end, because of the oligo (DT) primers used in the first-strand cDNA synthesis(40, 41). We also noted that there is pronounced difference in biases between the two batches of isotonic samples (Fig. 2A).

### Library quality control reduces technical artifacts

**Fig. 3:**
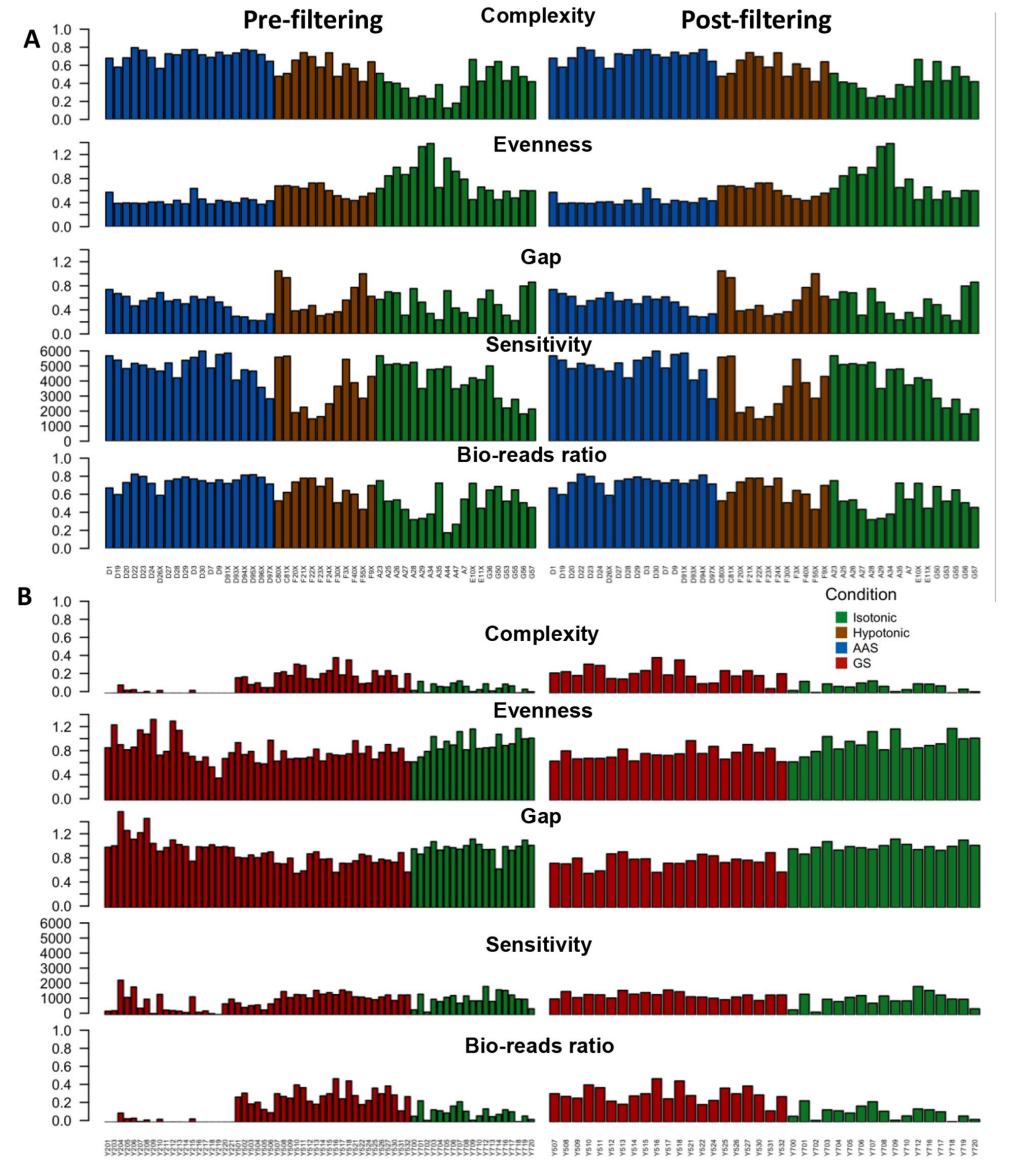
Quality control of scRNA-seq libraries using complexity, evenness, gap, sensitivity, and bio-reads ratio. In both A) and B), cells pre-filtering are shown on the left panel, and the cells post-filtering are shown on the right panel. **(A)** Batch 1 samples (51 samples pre-filter / 46 samples post-filter). (B) Batch 2 samples (66 samples pre-filter / 38 samples post-filter)

Since scRNA-Seq results are sensitive to multiple factors during library preparation, we evaluated each library for its quality by several assessment criteria: complexity, evenness of coverage, continuity of coverage, sensitivity, and bio-reads ratio (see Methods). The complexity of the libraries ranges from 0 to 0.80 with an average of 0.30 (Fig. 3A). There is significant difference between the complexity of the two batches, with samples of batch 2 showing lower complexity, indicating the presence of a strong bias in fragment amplification and insufficient sampling of mRNA molecules in the individual cells (Fig. 3A). This result is consistent with the considerably lower gene detection rate for batch 2 isotonic cells than for isotonic cells in batch 1 (Fig. 1A and E). Most libraries have less than one gap on average, though a few exhibits high number of gaps. In general, these measurements of library quality from different aspects are highly correlated (Fig. 3A). We filtered out low quality cells using a procedure as detailed in the Methods section. As shown in Figs. 3B and 84 of the 117 libraries passed the filters set by the five metrics. Clearly, after filtering, the distributions of the metrics are more uniform, most notably in the case of the GS treatment. Thus, our subsequent analyses were based on these 84 cells. Furthermore, we included ERCC spike-in in most of our samples as a quality control for assessing the accuracy of our gene expression quantification. The assessment of quantification quality was carried out using all bulk/single cells samples with spike-in rate of > 2%. It can be seen in **Supplementary Fig. S1A** that the expression levels (TPM) of spike-ins added in single-cell samples correlate well with the concentrations of the spike-ins (PPC = 0.842), indicating that our expression quantifications are reliable. Furthermore, we show that ERCC spike-in in bulk RNA-seq samples all show similar levels of correlation with known concentrations despite the different concentrations of spike-in added (**Supplementary Figs. S1B-S1G**). However, it is worth noting that as the concentration of added spike-ins increase, the quantified expressions show lower variance between samples as expected. We also applied the detection limit metric described in [40] to our spike-in data, using samples with at least 2% spike-in rate and having at least 8 different spike-in with non-zero expressions for this calculation. For these samples, the detection limits found were similar to the levels described in [40] for the SmartSeq2 protocol (Supplementary Fig. S1 H), indicating our procedures are reliable in terms of the ability to detect RNA molecules. All quality metrics for biological reads and spike-ins as well as additional quality metrics such as Mitochondrial gene % and Ribosomal RNA % are also included in **Table S1, S2** in **Supplementary File 1.**

Pearson correlations were also calculated between our samples post filtering and those of previous studies described in the Methods section (Supplementary Fig. S1 I)[41–43]. Although these studies performed single cell RNA-seq on a slightly different cell line (BY4741) and used different protocols in their sample preparation, our batch 1 isotonic cells were able to achieve a mean Pearson correlation of 0.59 (max: 0.91, min:0.26) across all cells under a similar unstressed condition, indicating that the transcriptomic profiles of our batch 1 isotonic yeast cells are similar to unstressed yeast cells from these previous studies. Moreover, the study of [43] utilized a slightly modified Smartseq-2 protocol, and thus our batch 1 isotonic cells correlate very well with their 2-hour cells (Pearson correlation max: 0.91, min:0.45, mean: 0.70). Differences in the degree of correlation is likely due to differences in sample preparation protocol, yeast strains used (S288C vs. BY4741), and slight variations in the condition of the cells. On the other hand, our AAS and hypotonic cells had much lower correlations. Isotonic samples from Batch 2 correlated poorly to all the studies due to strong batch effects and technical artifacts in these samples, (Pearson correlation max: 0.45, min:0.04, mean: 0.23). It is worth noting that despite the poor correlation of Isotonic Batch 2 samples with unstressed cells from previous datasets, the degree of correlation was still consistently higher than the correlation between GS samples (also from Batch 2, Pearson correlation max: 0.33, min:0.006, mean: 0.12) and the unstressed cells from previous datasets (Supplementary Fig. S1 I), again indicating that poor correlation likely originates from batch effects and that inherent biological differences caused by stress are still preserved in the transcriptomic profile within the same batch. Due to the presence of these strong batch effects, the technical differences between the two batches are too significant for aggregated analysis, thus subsequent analyses were performed in a batch-specific manner: isotonic vs. AAS and isotonic vs. hypotonic comparisons were conducted with cells from batch 1, while isotonic vs. GS comparison was performed with cells from batch 2.

### AAS and GS treatments induce distinct transcriptomes while hypotonic stress does not

**Fig. 4:**
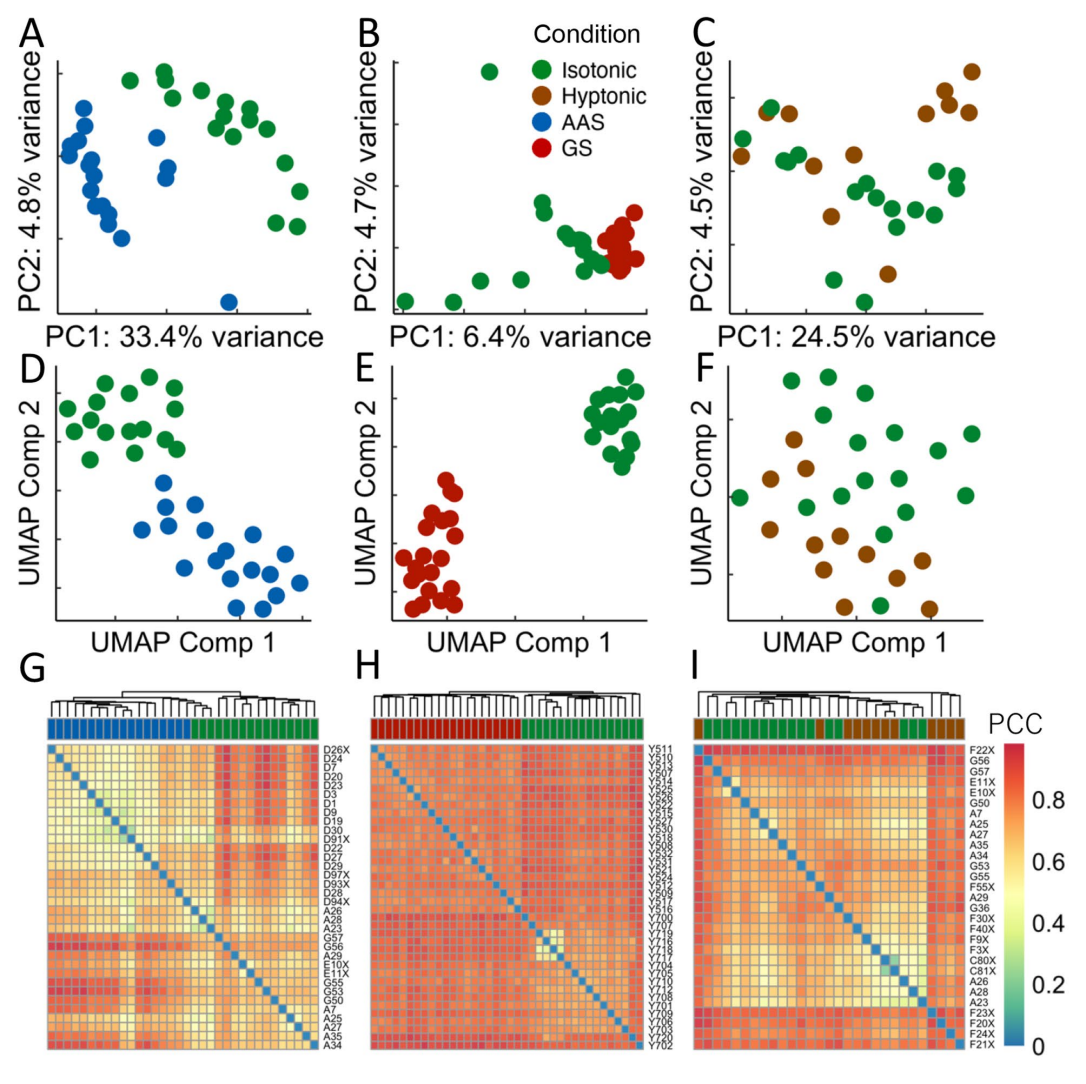
Comparison of transcriptomes of the cells under different stress conditions with those of cells under the isotonic condition. A, B, C. Visualization of the cells under AAS (A), GS (B) and hypotonic stresses (C) in comparison with cells under the isotonic condition using the first and second PCA components of their transcriptomes. **D, E, F.** Visualization of the cells under AAS (D), GS (E) and hypotonic (F) stresses in comparison with cells under the isotonic condition using the first and second UMAP components of their transcriptomes. **G, H, I.** Heatmaps of hierarchical clustering of the cells based on the Pearson correlation coefficients (PPC) of their transcriptomes using complete linkage. Color code shown in (B) applies to all the figures

To characterize the transcriptome features of the cells under different treatments, we compared the transcription levels of the 5,419 genes (**Table S3** in **Supplementary File 2**) that were expressed in at least 20% of the cells using principal component analysis (PCA) (Fig. 4A ~ 4C), uniform manifold approximation and projection (UMAP) (Fig. 4D ~ 4F) and hierarchical clustering (Fig. 4G ~ 4I). Clearly, cells under either AAS (Fig. 4A, D and G) or GS (Fig. 4B and E H) treatments display distinct transcriptomes from cells under the isotonic condition, suggesting that both AAS and GS triggered relevant gene regulatory pathways in the cells. However, cells under hypotonic stress did not separate well from isotonic cells in PCA (Fig. 4C), UMAP (Fig. 4F) and Hierarchical Clustering (Fig. 4I), suggesting that the hypotonic stress did not significantly alter gene transcription in the cells.

**Fig. 5:**
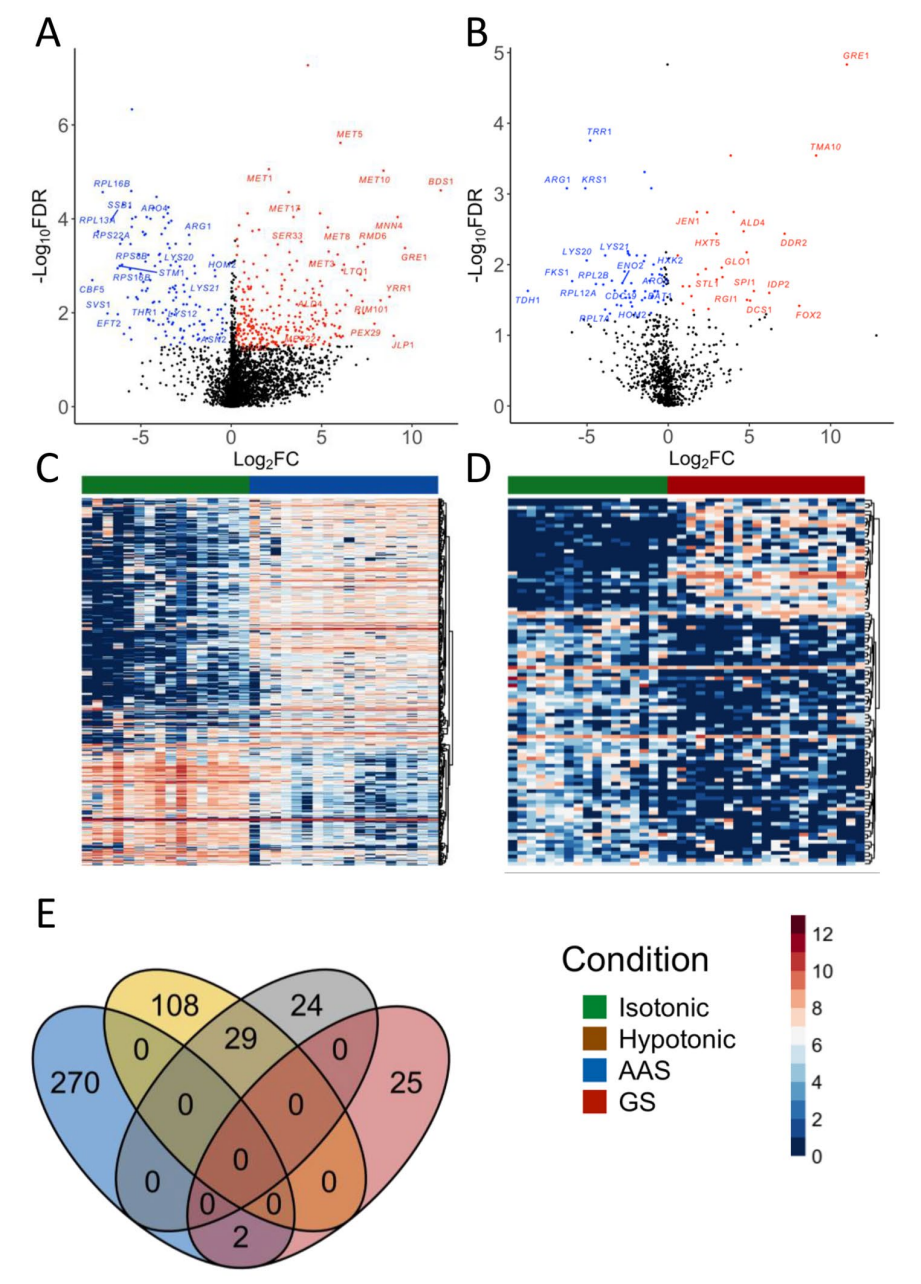
AAS and GS induce distinct gene expression patterns. A. Log2FC vs -Log10 (adjusted p-values) volcano plots of DEGs under AAS. B. Log2FC vs -Log10 (adjusted p-values) volcano plots of DEGs under GS. C. Heatmap of the expression levels of DEGs under AAS. D. Heatmap of the expression levels of DEGs under GS. Bars on the top of heatmaps indicate the treatments of cells according to the colors shown in the legend. The intensity of the heatmaps is colored according to Log2(TPM+1) values. E. Venn diagram of AAS upregulated, AAS downregulated, GS upregulated and GS downregulated genes

We next identified differentially expressed genes (DEGs) between cells under AAS, GS and hypotonic stresses to those under isotonic treatment using MAST [44] (FDR < 5%, absolute (Log_2_(fold-change (FC)) > 0.5). We identify 409 genes that were significantly differentially expressed under AAS relative to the isotonic condition (**Table S4** in **Supplementary File 2**), of which 137 were down regulated while 272 were up regulated (Fig. 5A C). Under GS, 80 genes were significantly differentially expressed relative to the isotonic condition (**Table S5** in **Supplementary File 2**), of which, 27 and 53 were significantly upregulated and down regulated, respectively (Fig. 5B and D). Contrastingly, no genes were found to be significantly differentially expressed in hypotonic condition relative to the isotonic condition. This is consistent with the results from the above PCA (Fig. 4C), UMAP (Fig. 4F) and clustering (Fig. 4I) analyses, where cells under isotonic and hypertonic conditions are indistinguishable. In our experiments, cells under all treatments (AS, GS, hypotonic and isotonic) were exposed to 1 M sorbitol solution for about one hour after being harvested from the log-phase growth in YPD in a procedure to remove the cell wall. Subsequently, to induce hypotonic shock, isotonic cells were exposed to a sorbitol lacking solution for at least half hour before being collected, at which point the cells had adapted to the hypotonic environment according to the earlier study [2].

The 272 upregulated genes under AAS are enriched for Sulfur metabolism and Sulfur amino-acid pathways, such as MET*1/3/5/8/10/17/22* as well as for the general response *GRE1* (Fig. 5A). The 137 down-regulated genes are enriched for ribosomal and translation functions, 77 of which encode components of a ribosomal subunit. Moreover, we found that genes involved in lysine (*LYS12/21/20*), threonine (*THR1, HOM2*), arginine (*ARG1)*, asparagine (*ASN2*) and aromatic amino acids (*ARO4*) biosynthesis are significantly downregulated (Fig. 5A). These results indicate that there may be a preferential synthesis of Sulfur amino acids when the cell was limited for all amino acids. A more recent study has found that methionine functions as an anabolic signal to induce global gene expression changes when in excess and that the presence of methionine can trigger the synthesis of other amino acids under AAS to sustain [45]. Furthermore, genes involved in the biosynthesis of glycine and serine (*SHM2*, *SER33)* were also found to be significantly upregulated. In this regard, glycine and serine have been known to be crucial one-carbon donor molecules in yeast and contribute to the synthesis of methionine [46].

**Fig. 6:**
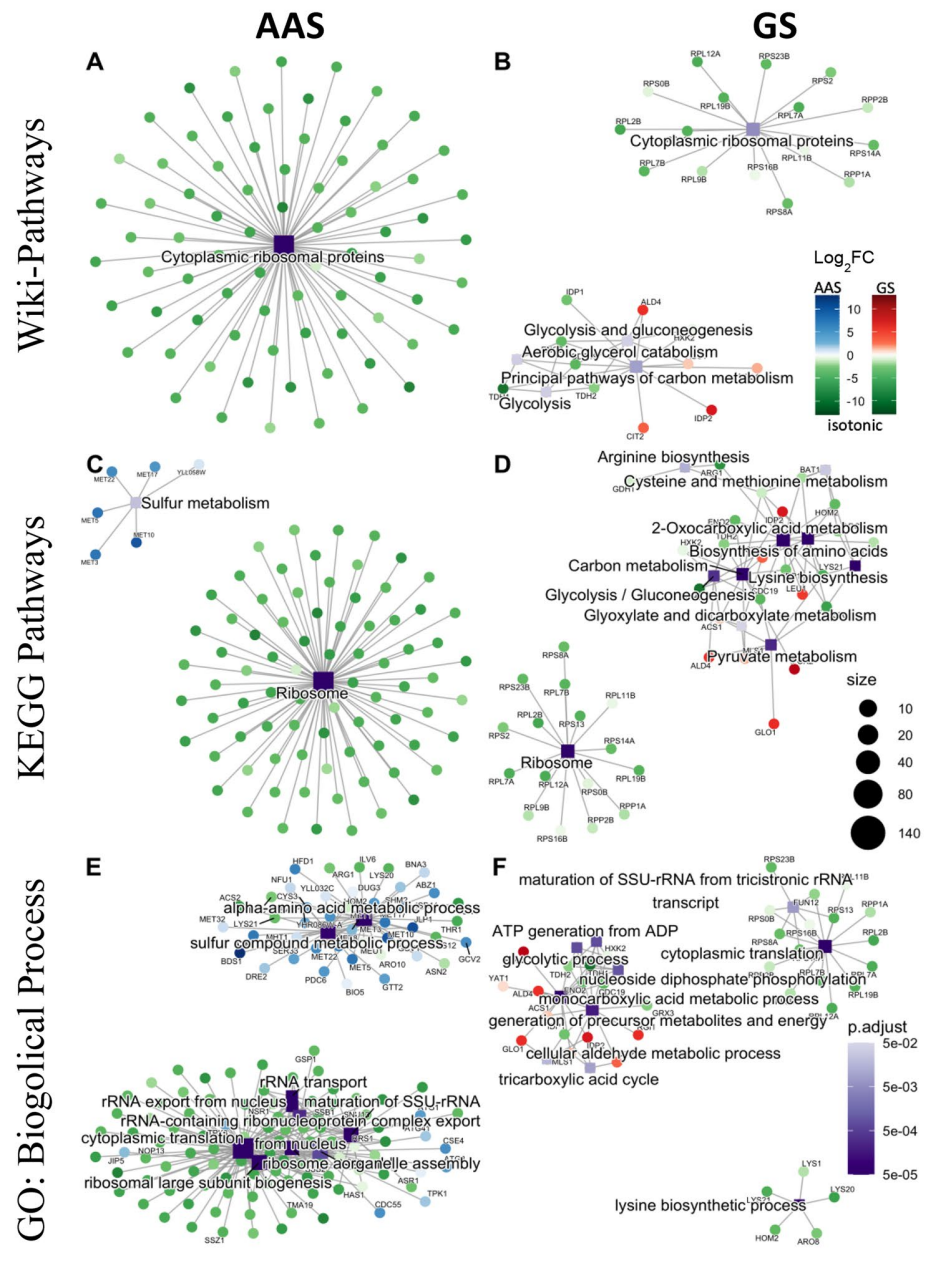
Enrichment of up- and down-regulated genes for known functional modules under AAS and GS. A, B. Wiki-Pathways enrichment under AAS (A) and GS (D). **C, D**. KEGG pathways enrichment under AAS (C) and GS (D). **E, F**. GO term biological process enrichment under AAS (E) and GS (F). Each square represents an enriched functional module. Color of a circle reflects the adjusted p-value while size of the square reflects the number of genes in the enriched functional module. Each circle represents a gene that is connected by an edge to its belonging functional module. Genes are colored by their Log_2_FC under the AAS or GS relative to the isotonic condition. False discovery rate (FDR < 5%) was controlled using the Benjamini-Hochberg procedure

Of the 53 downregulated genes under GS stress, 29 were also under-regulated under AAS stress (Fig. 5E). Specifically, 16 of the 53 downregulated genes encoded ribosomal subunits (Fig. 5B). There is also a down-regulation of several amino acid biosynthesis genes such as *LYS20/21, ARO8, BAT1, HOM2* and *ARG1* (Fig. 5B), possibly by Snf1p mediated repression of *GCN4* translation [47]. The other down-regulated genes are mainly involved in carbohydrate metabolism. For example, hexokinase isoenzyme 2 (Hxk2p) showed decreased expression (Fig. 6E). Previous studies have shown that under GS, activation of Snf1p prevents the interaction between Mig1p and Hxk2p, leading to de-repression of carbon-source responsive element (CSRE) containing genes involved in the use of alternate carbon [48]. The reduction in *HXK2* transcription might be a possible alternate mechanism to derepress CSRE containing genes. Our results also show decreased expression of *ENO*2 and *CDC*19 (Fig. 6E). Eno2p and Cdc19p catalyze the last two steps in glycolysis, respectively, where 2-phosphoglycerate is converted by Eno2p to phosphoenolpyruvate, which is then converted to pyruvate by Cdc19p [49]. It has been found that under GS, the activity of Cdc19p is quickly [50]. Furthermore, mRNA levels of *CDC*19 and *ENO*2 are also reduced through phosphorylated eIF4G regulation of the degradation [51].

The 27 upregulated genes under GS are glucose-repressed or involved in the use of alternate carbon resources (Fig. 5C). For instance, the expression of *HXT5*, *STL1* and *JEN1* (Fig. 5C) were significantly increased (**Table S5 in Supplementary File 2**), whereas *HXT5* encodes a hexose transporter that has affinity for glucose only under GS [52]; *Stl1*, a high affinity glycerol importer [53] and *JEN1*, a high affinity symporter for alternate carbon sources such as lactose, pyruvate, and acetate [54, 55]. Moreover, the expression of *FOX2* (Fig. 5C), which encodes a multifunctional enzyme in the peroxisomal fatty acid beta-oxidation pathway was significantly up-regulated (Fig. 5C). It has been shown that *FOX2* also is activated in autophagy [56]. These results are consistent with the earlier notion that under GS when the extra-cellular environment provides no other carbon resource, in order to survive the cells might need to recycle cytoplasmic components (bulk autophagy) and utilize an alternative source of energy (most likely metabolism of lipids) from within the cell [57].

Interestingly, we only found two genes *ALD*4 and *GRE1* to be upregulated under both GS and AAS stresses (Fig. 5A, B and E). Previous studies have shown that the expression of *GRE1* is controlled through the HOG pathway and its transcripts accumulate under osmotic, ionic, heavy metal, heat shock and oxidative stress [21, 58]. Our finding that *GRE1* was induced significantly under both AAS and GS conditions suggests that it may play a role in nutrient-limiting stress responses as well. *ALD4* encodes mitochondrial acetaldehyde dehydrogenase, which is necessary for the growth of yeast on ethanol [59]. Although it is known that *ALD*4 is glucose repressed, our finding that it was also significantly upregulated under AAS stress suggests that *ALD4* might play a role in general stress response.

The DEGs are also significantly enriched for relevant Wiki-pathways, Gene Ontology (GO) biological processes and KEGG pathways. Specifically, down-regulated genes under both AAS and GS stresses, are enriched for translation-related Wiki-pathways (Fig. 6A and B), GO terms (Fig. 6E and F) and KEGG pathways (Fig. 6 C and 6D). Up-regulated genes under AAS stress were enriched significantly for Sulfur metabolism (Fig. 6 C) and alpha-amino acid biosynthesis (Fig. 6E). Both up- and down-regulated genes under GS were enriched for Wiki- (Fig. 6B) and KEGG (Fig. 6D) pathways and GO terms biological processes (Fig. 6F) related to carbon metabolism, pyruvate metabolism, gluconeogenesis, and tricarboxylic acids cycle. Notably, under GS, DEGs were enriched for GO terms related to biosynthesis of lysine, arginine, cysteine, and methionine (Fig. 6F), and most of these genes showed decreased expression, indicating that glucose limitation shut down the synthesis of these amino acids.

### Motifs found in the upstream regions of the DEGs reveal possible gene regulatory networks

To reveal possible gene regulatory networks responsible for the distinct expression patterns of gene induced under AAS and GS stresses, we identified possible TF binding sites (TFBSs) in the upstream regions of the DEGs under AAS and GS. We found six and eight motifs in the upstream regions of downregulated and upregulated genes under AAS, respectively. TomTom [60] matched these six and eight motifs to known motifs of 14 and 23 TFs, respectively, in the Yeastract database **(Table S10** in **Supplementary File 3)**. Such multiple hits are understandable, since it is well-known that TFs of the same protein family recognize highly similar motifs [61, 62]. These TFs include amino acid starvation response TFs Gcn4p and methionine biosynthesis regulators such as Met4p, Met31p and Met32p. The details of the identified motifs in the upstream regions of the DEGs under AAS are shown in **Table S6** in **Supplementary File 3.** Moreover, we identified 12 motifs for 23 TFs and 10 motifs for 27 TFs in the upstream regions of down- and upregulated genes under GS, respectively. These TFs are involved in alternative carbon source utilization, such as Pdr3p, Ert1p, Sip4p and Aaca1p. The details of the identified motifs in the upstream regions of the DEGs under AAS are shown in **Table S7** in **Supplementary File 3**.

**Fig. 7:**
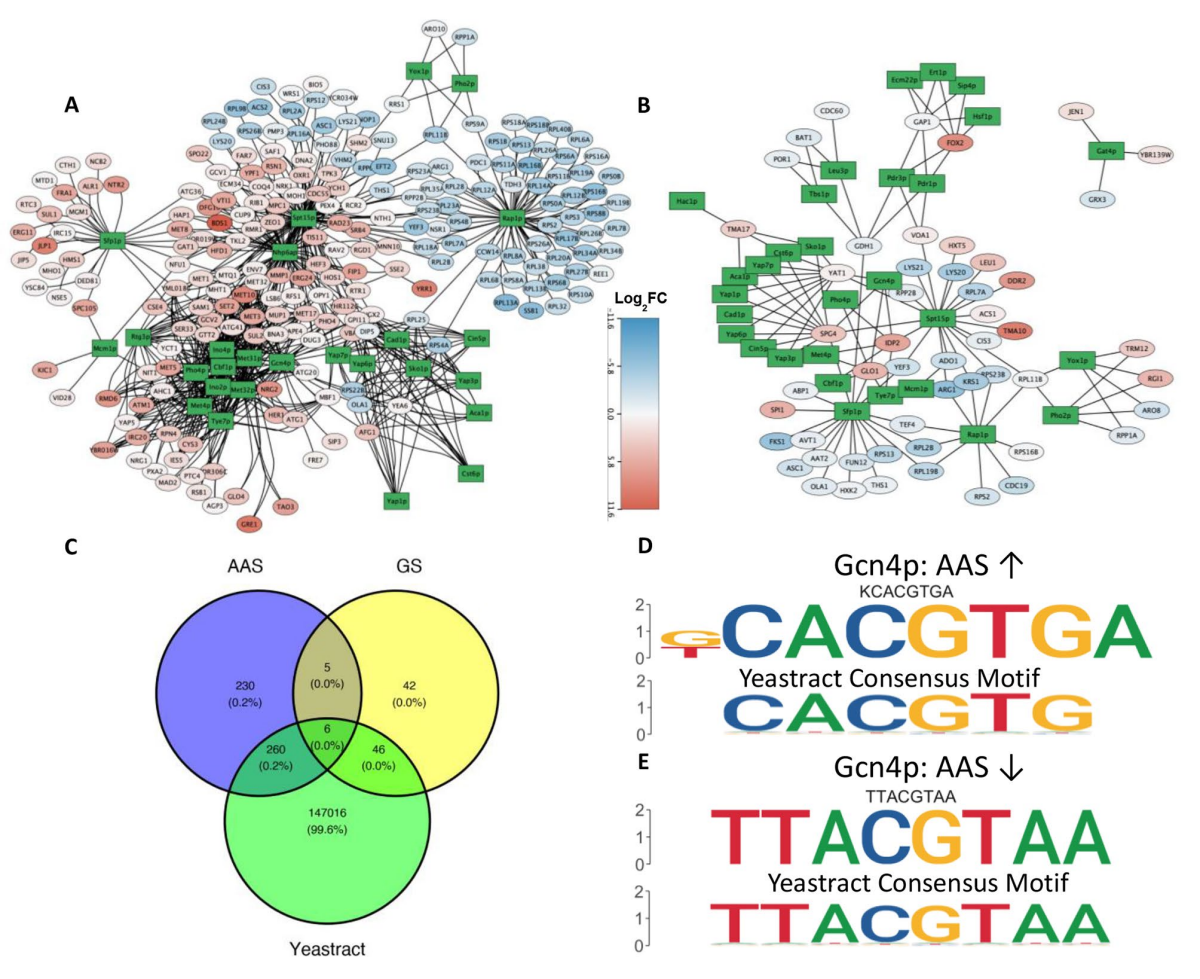
Inferred gene regulatory networks for stress-induced transcriptional changes. (A) Putative gene regulatory networks for the DEGs under AAS. (B) Putative gene regulatory networks for the DEGs under GS. TF genes are shown as green rectangles and non-TF genes as ovals. A TF and its inferred target gene are connected by an edge. Upregulated genes are colored red and downregulated genes blue. (C) Venn diagram of predicted TF-gene relationships and known relationships on the Yeastract Database. D, E. Different motif usages of Gcn4p in AAS for upregulated genes (D) and for downregulated genes (E)

Based on the identified TFBSs and putative cognate TFs, we constructed gene regulatory networks for the DEGs under AAS consisting of 594 putative regulatory relationships between 26 TFs and 218 genes (Fig. 7A, for the details see **Table S8 in Supplementary File 3**) and for DEGs under GS consisting of 114 putative regulatory relationship between 31 TFs and 55 genes (Fig. 7B, for the details see **Table S9 in Supplementary File 3**). Most of these inferred regulatory relationships are supported by existing data, while others might be novel findings. More specifically, 266 and 52 of our predicted TF-gene relationships under AAS and GS, respectively, are documented in the Yeastract database, while 235 and 47 of our predicted TF-gene relationships under AAS and GS, respectively, might be novel regulatory relationships (Fig. 7C). For examples, we confirm multiple ribosomal protein genes such as *RPL16B/13B/18B, EPS0B/1B/18A* etc. (for the complete list, see **Table S8 in Supplementary File 3**) that are regulated by TF Rap1p under both AAS and GS(Fig. 7A and B), which has been shown to control the expression of ribosomal genes [63]. We identified Spt15p binding sites in the upstream regions of multiple upregulated genes such as *HAP1, CDC55, HFD1*, etc. and downregulated genes such as *LYS20, LYS21, ACS2* etc. (**Table S6 in Supplementary File 3**) under AAS (Fig. 7A), and Spt15p is known for binding the TATA box in the promoters of many of these genes [64]. We found binding sites of TF Sfp1p in upstream regions of upregulated genes (*JLP1, SUL1*, etc.) under AAS (Fig. 7A). Interestingly, two of the inferred target genes of Sfp1p, *SUL1* and *JLP1* (Fig. 7A), both are involved in the uptake of Sulfur, have not been documented in previous research, thus, might be novel findings. Interestingly, while we inferred that Sfp1p mainly upregulated genes under AAS (Fig. 7A), it mainly down-regulate genes under GS (Fig. 7B). We show that *JEN*1 might be a target gene of Gat4p under GS, although it has been reported that *JEN1* was indirectly regulated by Gat4p [65]. We also found that *GRX3*, coding a glutathione-dependent oxidoreductase that protects the cell from oxidative damage [66], and *ATG42* (*YBR139W*), coding a vacuolar serine-type carboxypeptidase involved in the proteolytic processing and final steps of autophagy in [67], might be novel target genes of GAT4. While we were able to confirm that Pdr1p and Pdr3p were regulators of *FOX2* under GS (Fig. 7B) [68], we also inferred that Ert1p might be a novel regulator of *FOX2*, sharing a similar binding site as Pdr3p (**Table S7 in Supplementary File 3**).

Interestingly, some TFs are predicted to upregulate some genes and downregulate some other genes under the same condition. We thus examined whether the motifs found in upregulated genes and downregulated genes differed for such TFs. As shown in **Table S10 in Supplementary File 3**, most such TFs bind similar binding sites of the same motif in the upstream regions of both upregulated genes and downregulated genes. The different regulatory effects might have resulted from different locations of the binding sites and/or with different cooperative TFs [69, 70]. However, Gcn4p recognizes quite distinct two motifs in the upstream regions of upregulated genes (Fig. 7D) and downregulated genes (Fig. 7E) under AAS, suggesting different regulatory machineries of Gcn4p for upregulated genes than for down-regulated genes involved in amino-acid synthesis, a phenomenon that has been previously reported [70, 71].

Furthermore, we attempted to corroborate the putative regulatory relationships through a correlation analysis between the TFs and predicted target DEGs. In brief, the mean correlation between a TF and its predicted targets is tested for significance through a permutation test, see Methods section. The results showed that the expression of some TFs did not significantly correlate with its targets, indicating post-transcriptional regulation mechanisms of these TFs under stress, while some exhibit significant correlation, indicating regulation of those TFs at a transcriptional level. Notably, Met31p/Met32p/Cbf1p are known to be regulators for over 4,000 genes in Yeastract, however a permutation test finds all the three TFs significantly correlated with predicted targets under AAS (**Supplementary Fig. S2A**). Correlation between Met4p and its targets was also found to be significantly correlated with its DEG targets. These results seem to indicate that the response to AAS induces a transcriptional change in TFs that govern methionine biosynthetic genes. Gcn4p on the other hand was not shown to be significantly correlated with its predicted target DEGs, this reaffirms earlier studies showing Gcn4p to be controlled in a translational manner [16]. Interestingly, Gcn4p is shown to be significantly correlated with its target TFs under GS. GS is also known to induce Gcn4p activation via a translational mechanism through Gcn2p, however specifics of the process differ from that under AAS [72]. Our results indicate that Gcn4p may also be transcriptionally regulated in response to GS (**Supplementary Fig. S2 B)**.

### TFs contribute to cellular variability in a post-transcriptional manner

We also seek to elucidate whether the variability observed between single-cell transcriptomes of the same condition can be attributed to variation of transcriptional regulatory machineries such as variation of TF expression levels. Our single cell study naturally provides such an advantage over the conventional population-based study, in which minute differences in transcriptional regulation are obfuscated by the pooling of thousands of cells. Using cells under the same conditions (AAS, Hypotonic and Isotonic Batch 1 for their better quality), we performed hierarchical clustering (see Methods.) of genes that are expressed in at least 10% of all cells. Though not all clusters showed significant enrichment for regulating TFs, many clusters were able to enrich for TFs, indicating genes associated with the same TFs exhibit similar patterns of variation under the same conditions, suggesting biological variability between cells under the same conditions is due to the transient differences in TF activation states, and such variabilities can possibly be utilized to discover regulatory relationships. (**Supplementary Figs. S3A, S3B, S3C).** Interestingly, the enriched regulating TFs are rarely present in their respective clusters, likely due to delayed effects of TF induced activity and target genes expression change, and post-transcriptional activation of TFs.

## Discussion

Recently, scRNA-seq has become a powerful tool to address important biological problems including cell type identification, understanding mechanisms of gene transcriptional regulation and characterization of functionally related genes at the single cell level. Despite many applications of scRNA-seq to the most well-studied eukaryotic organism, scRNA-seq applied to the budding yeast are rare, likely due to the more technical demands associated with a tough cell wall and very low mRNA contents when preparing scRNA-seq libraries for a yeast cell. In this study we overcame the technical obstacles by adapting an earlier full-length scRNA-seq method [73, 74], and profiled the transcriptomes of single yeast cells in isotonic, nutrients-rich medium and under three stress treatments hypotonic, AAS and GS. However, to remove the cell walls, we inevitably exert stresses to the cells by exposing them to water and softening medium to facilitate subsequent enzymatic cell wall digestion. To minimize the impacts on the cells, we equilibrated the cells in the isotonic, nutrients-rich spheroplast medium at 30 °C for 60 min when digesting the cell with Zymolyase 100T, followed by 2 × 5-min washes of the cells in the spheroplast medium. The transcriptional responses of the cells under subsequent stress treatments are similar to those seen in previous studies, indicating that our cell wall removal procedure had little effects on the cells.

Though scRNA-seq improves upon previous transcriptomics quantification methods, cell-to-cell variation may be confounded by factors such as cell size, state and technical factors that arise during sample preparation. The scRNA-seq libraries in this study exhibited varying degrees of technical variation and batch effects. In particular, there exists significant technical differences and batch effects between our batch 2 and batch 1 samples, as shown in both QC metrics and correlation with previous studies. The lower quality of samples in batch 2 could be attributed to various technical factors. Therefore, a careful quality control is critical before any formal analysis to minimize the effects of technical variability. To this end, we filtered out low quality libraries using a stringent QC procedure based on five metrics, and only performed comparisons between transcriptome collected from the same batch. We show that this QC procedure was able to not only enhance the reliability of our analysis but also yield more biologically meaningful results.

Another critical aspect of inference from scRNA-seq data is the depth of read coverage, which has an effect on the number of genes that can be detected as has been noted in cell population based libraries as well [75]. To evaluate the effect of read depth, we prepared a series of diluted bulk RNA libraries, starting from 5 pg which is near the lower bound of mRNA levels in small single cells. From the saturation analysis using the varying number of reads randomly sampled from the 5 pg bulk library (Fig. 1), we deduced that a minimal sequencing depth of 4 × 10^6^ reads is required to detect almost all genes that can be quantitatively characterized at this input quantity of mRNA. Cells under a certain treatment exhibited higher correlation of transcription levels, while different treatments led to distinct transcription of relevant genes in response to the treatments. This indicates that transcriptome analysis at the single-cell level is biologically meaningful regarding stress conditions.

Our observation that the isotonic and hypotonic cells cannot be clearly differentiated using their transcriptomes is consistent with a previous study using microarray to characterize gene expression on diverse environmental transitions, which found that when cells were transferred from standard isotonic to hypotonic solution, the change in the expression of the genes involved in environmental stress response is only transient [2]. Consequently, our DEG analysis was not able to find significantly differentially regulated genes, suggesting that gene expression levels were indeed back to isotonic conditions.

As expected, individual cells under the same treatment show varying levels of variation due to intrinsic biological noise and unavoidable technical noise [36], though the latter has been largely reduced by our QC procedure. However, both PCA/UMAP and DEG analyses revealed larger variability among single cells under AAS vs. isotonic and GS vs. isotonic treatments. Under AAS stress, we observed significant down-regulation of translation related genes such as those encoding ribosomal proteins, consistent with the earlier [2], where ribosomal genes were found to be repressed during general stress response. More genes were found to be upregulated, particularly, those involved in the methionine and cysteine biosynthetic pathways. Previous studies have shown that the AAS-induced response in yeast cell could be activated under the depletion of even a single type of amino [16, 76]. In this study, the yeast cells were transferred to a YPD medium without any type of amino acids. Our results show that when depleted of all amino-acids, there was a very strong transcriptional response of genes responsible for the synthesis of Sulfur-containing amino acids. In this regard, methionine, one of the two amino acids containing Sulfur, has been found to be an integral part of signaling pathways involved in the inhibition of autophagy, the regulation of tRNA thiolation that controls overall metabolic state, and cell proliferation [45, 77]. Beyond its role as the initiation amino acid, methionine is also involved in increasing translation capacity through controlling upstream regulators in the TORC pathway [77]. Our finding that the DEGs under AAS stress are significantly enriched for pathways involved in Sulfur metabolism and Sulfur-containing amino acid biosynthesis, provides a different perspective to the earlier results. It seems that under the limitation of all amino acids, apart from a drastic reduction in ribosomal and translation activity, the yeast cells preferentially synthesize methionine first as a mechanism to induce the synthesis of other amino acids and prepare for the restart of anabolism once conditions are viable again.

Under the GS stress, there is also a significant down-regulation of translation and ribosomal encoding genes, consistent with early [1]. Reduced expression was also found in genes involved in the repression of other genes in the presence of glucose. Genes involved in biosynthesis of multiple amino acids were also found to be downregulated. The upregulated genes are mostly involved in the general stress response and processes related to the utilization of alternate carbon resources such as gluconeogenesis, hexose transporters and energy metabolism [41–46].

By identifying putative TFBSs in the upstream regions of the DEGs under AAS and GS, we were able to confirm known TF-target gene relationships as well as potential novel ones. Modes of regulation also seems to be different for shared TFs between different stresses, such as the case for Sfp1p, which is mainly associated with upregulated genes under AAS and downregulated genes under GS.

Enrichment of TFs in clusters of expressed genes was able to provide an explanation for the observed cellular variability between cells of the same condition. We showed that, with samples of decent quality, it is possible to identify gene modules regulated by the same TF using only intrinsic biological variability without the introduction of perturbation. This is likely a result of stochastic variation in TF activation differences between cells of the same condition and the propagation of such variation or noise in gene regulatory networks [78]. This finding provides further [78, 79], supporting the idea of using cellular variability/noise to study regulatory mechanisms.

## Conclusion

We have demonstrated the possibility of sequencing single-cell full-length mRNA transcriptomes in small *S. cerevisiae* cells, generated a scRNA-seq data set in the organism under isotonic, hypotonic, AAS and GS conditions. Our analysis of these data provided insights into genes that are differentially expressed in *S. cerevisiae* under the stressors. Our results align closely with early studies into the stress response of *S. cerevisiae*, while also providing unique findings. We highlight the preferential increase in the expression of genes for the biosynthesis of methionine over other amino acids when all amino acids are absent. In addition, gene regulatory networks underlying the transcriptional responses were highlighted through the analysis of TFBSs for each of the DEGs. The DEGs and gene regulatory networks uncovered in this study provide a single cell based transcriptomic view for future studies of stress-induced responses in this model organism. Moreover, we showed that biological variability between cells under the same conditions due to stochastic events can be utilized to discover regulatory relationships.

## Methods

### Experimental methods

#### Cell culture and spheroplasts preparation

A monoclone of the yeast strain S288C (ATCC) was selected using a YPD based agar (10% yeast extract, 20% peptone, 2% glucose and 20% agar) petri plate and stocked at -80 °C until use. To wake up cells, 30 µl thawed yeast stock inoculated in 3 ml YPD medium (1% yeast extract, 2% peptone and 2% glucose) was incubated overnight at 30 °C and 250 rpm. Cells were then expanded at 30 °C and 250 rpm after a 1:50 dilution in the YPD medium until mid-logarithmic phase (OD_600_ between 0.5 and 0.8). Five OD unit (ODU) cells were collected by centrifugation (500 g, 5 min) at room temperature. The cells were resuspended in autoclaved water and collected by centrifugation (500 g, 5 min) at room temperature. The cells were then resuspended in the softening medium (100 mM Hepes-KOH, pH 9.4, 10 mM Dithiothreitol) and incubated in room temperature for 15 min. The cells collected by centrifugation (500 g, 5 min) at room temperature were then resuspended in the Spheroplasts (S) medium (1× YNB, 2% glucose, 1x amino acids, 50 mM Hepes-KOH, pH 7.2, and 1 M sorbitol) [80] to a concentration of 5 ODU/ml. Zymolyase 100T was added to the spheroplasts suspension to a final concentration of 2 µl/ODU, followed by 60 min incubation at 30 °C to remove the cell wall and equilibrate cells to an isotonic, nutrient-rich condition. After two washes in the S medium by centrifugation (500 g, 5 min) at room temperature, spheroplasts were re-suspended to 5 ODU/ml in the desired treatment solution: amino acids starvation (AAS): S medium (with 1.0 M Sorbitol) without amino acid; glucose starvation (GS): S medium (with 1.0 M Sorbitol) without glucose; hypotonic: S medium without sorbitol; isotonic condition: S medium with 1 M sorbitol. Cells were exposed to the treatment for 0.5 ~ 2.0 h before manual harvest.

#### Single cell harvest

0.5 mL of the spheroplasts were placed on a poly-lysine coated circular cover slip (2 mm diameter) in a petri dish for 5 min at room temperature (23 °C). The cover slip was broken in the center with forceps, and a small piece of cover slip was transferred to a 30 µl perfusion chamber, which was constantly perfused by a desired solution by gravity feeding. The solution change time in the chamber was about 20 s. Single cells were harvested using a path clamp electrode pipette using a micromanipulator (ROE-200, Sutter) under an inverted microscope (Olympus 1 × 71) placed on a vibration isolation table (TMC). A cell was harvested in less than 10 nl perfusion solution.

#### Single cell RNA-seq library preparation

Our method is based on [73, 74] with modifications to prepare multiplex sequencing libraries using Illumina Nextera XT Kit. Briefly, a harvested cell was quickly transferred using a home-made microinjection system to a 200 µl Eppendorf tube containing 4 µl cell lysis buffer (0.9× PCR Buffer II, 3 mM MgCl_2_, 0.45% NP40, 4.5 mM DTT, 0.18 U/µl SUPERase-In, 0.36 U/µl RNase Inhibitor, 12.5 nM AUP1 primer, 2 mM dNTP). In most single-cell samples, 0.1 µl (1:2.5 × 10^4^ dilution) ERCC spike-in mRNA (Thermo Fisher) was added. The cell was lysed at 70°C for 90 sec, then placed on ice and stored at -80°C until use. A cell lysate was thawed on ice, and 1 µl reverse transcription mix was added (13.2 U/ µl SuperScript III Reverse transcriptase, 0.4 U/µl Rnase Inhibitor, and 0.07 µg/µl T4 gene 32 protein). The first strand cDNA was synthesized by incubating the tube at 50°C for 30 min, followed by inactivation of the reverse transcriptase at 70°C for 10 min, and then the tube was cooled on ice. Free AUP1 primers were removed by adding 1 µl ExoSAP (Affymetrix) to the tube and incubating at 37°C for 15 min, followed by inactivation of the ExoSAP at 80°C for 15 min. This step would leave the AUP1 sequences at the 5’-end cDNA intact. A polyA tail was then added to the 3’-end of the first strand cDNA by adding 6 µl TdT mixture (1× PCR Buffer II, 1.5 mM MgCl_2_, 3 mM dATP, 0.75 U/µl Terminal Transferase and 0.1 U/µl RNase H) and incubating at 37 °C for 15 min, followed by inactivation of the enzyme at 70 °C for 10 min. The resulting products (12 µl) were then divided into two equal portions (each 6 µl), and each was mixed with 19 µl second strand buffer (1× High Fidelity PCR Buffer, 2 mM MgSO_4_, 0.2 mM each dNTP, 0.3 µM AUP2 primer, and 0.1 U/µl high fidelity Platinum Taq DNA polymerase). The two tubes were subject to one PCR cycle (30 s at 95 °C, 2 min at 50 °C and 6 min at 72 °C) to synthesize the second-strand cDNA in the form of 5’-AUP2-T24-cDNA-A24-AUP1-3’. Nineteen µl PCR mixture (1× High Fidelity PCR Buffer, 2 mM MgSO_4_, 0.25 mM each dNTP, 2 µM AUP1 Primer, 2 µM AUP2 Primer, 0.1 U/µl Platium Taq DNA Polymerase High Fidelity) was added to each tube, which brings the volume of each reaction to 44 µl, and cDNA was amplified by 18 PCR cycles (98 °C for 5 s, 67 °C for 1 min and 72 °C for 6 min). The resulting cDNA from two reactions were combined (total 88 µl) and were further subject to 12 cycles of PCR with two duplicates, each with 2.4 µl sample and 87.6 µl PCR mixture (1× High Fidelity PCR Buffer, 2 mM MgSO_4_, 0.375 mM each dNTP, 1 µM AUP1 Primer, 1 µM AUP2 Primer, 0.1 U/µl Platium Taq DNA Polymerase High Fidelity). The products were then combined, and cDNA was revolved on a 1% ager gel (25 µl sample per lane). The band between 300 bases to the loading well was cut and cDNA was purified using a QIA quick gel purification Kit, followed by magnetic beads (GE Health) purification (10:7 sample to beads ratio). After quantification using a Bioanalyzer (Agilent High Sensitivity DNA Kit), the libraries were then prepared using an Illumina Nextera XT or TruSeq DNA Sample Preparation Kit according to the vendor’s guide. The libraries were sequenced on an Illumina HiSeq2000 or HiSeq2500 machine (100 base-paired reads). Bulk mRNA was also extracted from population spheroplasts under AAS using a yeast RiboPure™ RNA Purification Kit (Ambion). Different amount of purified bulk mRNA (5pg, 10pg, 20pg, 1,00pg, 1000pg and 10,000pg) were used to construct sequencing libraries in the same way as for single-cell libraries, with the exception that 0.1 µl ERCC spike-in mRNA (Thermo Fisher) was added to the lysis buffer, with a concentration of 1:5 × 10^5^, 1:2.5 × 10^5^, 1:1.25 × 10^5^, 1:2.5 × 10^4^, 1:2.5 × 10^3^ 1:2.5 × 10^2^ for the 5pg, 10pg, 20pg, 100pg 1,000pg and 10,000pg input RNA, respectively.

## Statistics and reproducibility

### Characterization of single-cell transcriptomes

The raw reads from FASTQ files of different lanes were first combined based on sample ID before being mapped to the *S. cerevisiae* reference genome (SGD R64-2-1) using STAR (version 2.5.2) [81]. STAR alignments provided soft clipping of possible adapter sequences at the ends of reads; thus, no prior trimming of reads was performed. Gene transcription levels were quantified using uniquely mapped reads in TPM(transcript per million mapped reads) by RSEM(v1.2.31) [82] and in raw counts by [83] (default parameters, using the formatted for DESeq2 count files). Principal components analysis (PCA) and uniform manifold approximation and projection (UMAP) of the cells/samples were performed on the log_2_(TPM + 1) values.

### Library Quality Assessment

We adopted the following metrics to quantify the quality of the scRNA-seq libraries from each cell/sample. (1) The library complexity is defined as the number of distinct (unique) read’s start positions mapped to the genome [84]. To directly compare the library complexity of different libraries, we randomly sampled the same number of reads (one million) from each library. (2) The evenness is defined as the averaged coefficient of variation (CV) of the read coverage along each base-pair of the gene body [84]. Since transcripts of a low copy number are subject to uneven coverages, only the top 50% highly expressed genes are used in the calculation of this measure. (3) Continuity of coverage measures the number of gaps along the exons of a gene, where a gap is defined as a consecutive length of ≥ 5 bases without any reads mapped. The final gap measure is a weighted average of gaps in all the genes according to each gene’s expression in TPM values. (4) Sensitivity, which measures the number of genes detected with at least 5 reads in each sample. And (5) Bio-reads ratio, which measures the proportion of reads uniquely mapped to the organism genome. Sample quality control and filtering were performed slightly differently on the two batches of our libraries due to their different sequencing depth and quality: samples from **batch 1** were retained if the following thresholds were met: detection rate > 500, complexity > 0.1, gap < 0.5, evenness < 1.5; while samples from **batch 2** were retained if the following thresholds were met: num uniquely mapped reads > 10,000. All quality metrics apart from Sensitivity were calculated for spike-in reads for each sample independently as well. Metrics such as rRNA expression % and Mitochondrial gene expression % were calculated using TPM values, genes used in these calculations are included in Table S1, Supplementary File 1. An additional metric was calculated for spike-in reads based on the detection-limit procedure described in [40] using TPM expression of cells that had > 2% spike-in rate and at least 8 different spike-ins with non-zero expression. Pearson correlation was calculated between all samples that passed filtering and samples of previous [41–43]. Normalized expression data was obtained from GEO according to the authors, specifically, [42] and [43] was normalized with the median normalization used in DESeq2[85], and data from [41] was normalized with the SCnorm [86]. In order to select samples similar to the isotonic conditions used in our study, yeast cells aged 2-h were selected from the study of [43], samples labeled ‘Unstressed’ were selected from [41] and only samples of the BY4741 strain were selected from [42]. For each study, we normalized our data according to the specified normalization method and used gene features shared between our data and the selected dataset in the calculation of Pearson correlation.

### Differential expression analysis and GO/Pathway Enrichment

Analysis of differentially expressed genes was performed using MAST [44]. For each comparison, only genes expressed in at least 20% of samples were used for subsequent analysis. Counts per million (CPM) expression values (calculated with raw counts) were used as the input and all other parameters were left at default. Differential expression results were controlled for gene detection rate. Genes that met the criteria: FDR < 0.05 and absolute fold-change > 1.2 were labeled as differentially expressed. Enrichment analyses for Gene Ontology biological [87, 88], KEGG [89–91] and Wiki-[92] were carried out using clusterProfiler [93] based on the identified differentially expressed genes.

### Promoter and transcription factor binding Sites analyses

Upstream sequences (maximum length of either 1,000 bp or the entire upstream intergenic sequences) of differentially expressed genes (DEGs) were extracted from the *S. cerevisiae* reference genome (SGD R64-2-1). Motifs in the sequences were identified using ProSampler [94], which were then compared with known motifs in the Yeastract database [95] using TomTom [60]. This analysis was performed separately for upregulated genes and downregulated genes of each stress condition. TF-gene interactions from Yeastract were extracted for all genes filtering for interactions that were either documented or had gene expression evidence. When comparing our putative TF-gene relationships with TF-gene relationships from Yeastract, we omitted relationships involving Spt15p and Nhp6ap from our analysis as these TFs are known to bind TATA boxes and are also absent from TF-gene relationships from Yeastract.

Empirical correlation analysis between TF and predicted target DEGs was performed as follows:

Given a table of TF-gene interactions, for each TF:

Step 1: calculate the mean correlation C between TF and its interacting genes.

Step 2: let N be the number of predicted DEGs interacting with the TF, sample N genes from all genes and calculate mean of absolute correlations between the TF and N sampled genes.

Step 3: repeat step M (M > = 10000) times to generate empirical distribution of mean of absolute correlations.

Step 4: p-value = (Number of sampled mean of correlation values > S)/M

### Gene Clustering and TF Enrichment

For samples of the same condition and batch, absolute Pearson correlation distance (1-| Pearson correlation|) was calculated between gene TPM expression levels and used for hierarchical clustering using the hclust method in R with method set to ‘ward.D2’. Number of clusters was determined with R package NbClust (min.cluster = 2, max.cluster = 200)[96]. Each resulting cluster was then enriched for TFs using known Yeastract TF-Gene interactions using a hypergeometric test from R package [93]. Each cluster was then assigned with the most significant TF only if its Benjamini-Hochberg adjusted p-value < 0.05.

## Electronic supplementary material

Below is the link to the electronic supplementary material.

**Additional file 1. Figure S1.** ERCC Spike-in Expression (TPM) vs Known Concentration in samples with at spike-in rate > 2%. **Figure S2. **Spearman correlation significance test between predicted target DEGs and TFs. **Figure S3. **Hierarchical clustering of genes under each condition and enrichment of transcription factors for each cluster.

**Additional file 2. Table S1.** Summary of mapping results of the scRNA-seq libraries. **Table S2.** Summary of mapping results of the bulk RNA-seq libraries

**Additional file 3. Table S3.** Raw Counts of Samples and Genes used for DEG. **Table S4.** Summary of DEGs under AAS. **Table S5. **Summary of DEGs under GS.

**Additional file 4. Table S6.** Motifs found in the upstream regions of DEGs under AAS. **Table S7.** Motifs found in the upstream regions of DEGs under GS. **Table S8.** Inferred TF-target gene relationships under AAS. **Table S9.** Inferred TF-target gene relationships under GS. **Table S10.** Summary of Number of DEGs and Binding Motifs for each TF.

## Data Availability

The scRNA-seq data generated are available at GEO with access number GSE201387. All other data are available in the Supplementary files.
