# Supplementary material for: Transcriptomic changes in single yeast cells under various stress conditions
Source: BMC Genomics. 2023 Feb 24;24:88. doi: 10.1186/s12864-023-09184-w (PMC9960639; doi:10.1186/s12864-023-09184-w)
Supplement: Supplementary file 1 — Additional file 1. Figure S1. ERCC Spike-in Expression (TPM) vs Known Concentration in samples with at spike-in rate > 2%. Figure S2. Spearman correlation significance test between predicted target DEGs and TFs. Figure S3. Hierarchical clustering of genes under each condition and enrichment of transcription factors for each cluster. [file 12864_2023_9184_MOESM1_ESM.docx]

**Supplementary Fig S1** **ERCC Spike-in Expression (TPM) vs Known Concentration in samples with at spike-in rate > 2%.** A-F: 5pg, 10pg, 20pg, 100pg, 1ng, 10ng bulk-RNAseq sample ERCC expression vs known concentration(molecule/μl). G: single cell RNAseq ERCC expression vs known concentration (molecule/μl). H: Detection limit metric described in (1). I: Pearson correlation with previous datasets(2-4) colored by condition and batch, black bars in box plot represent mean Pearson correlation.


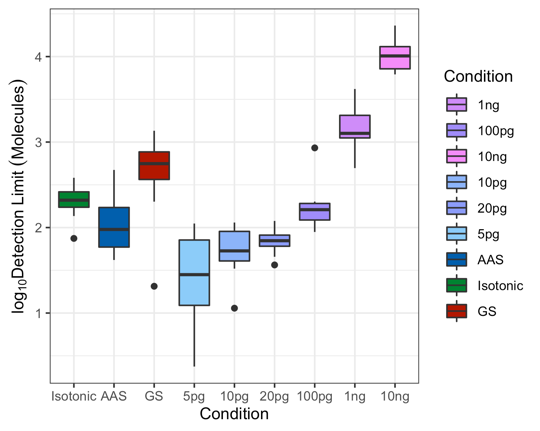

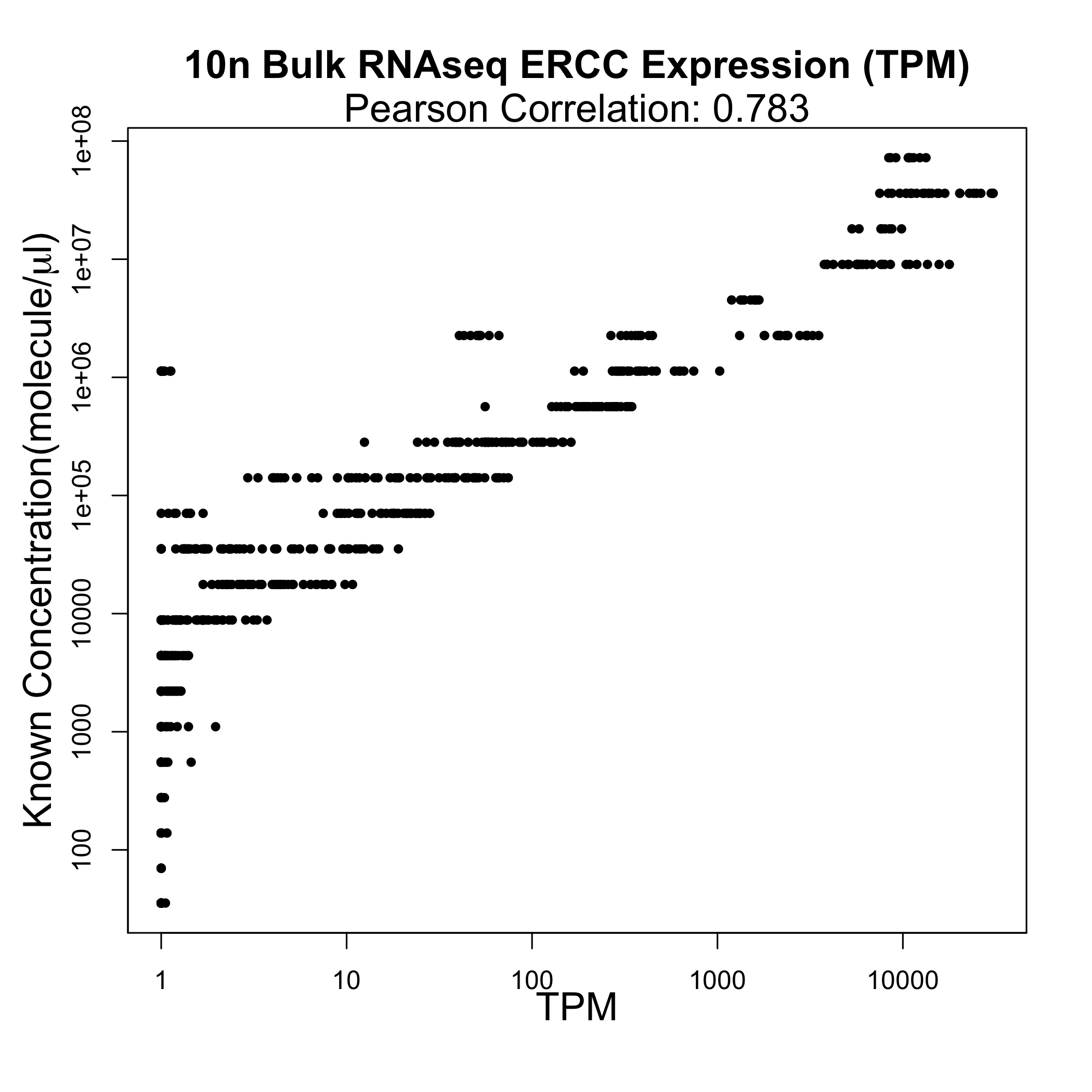

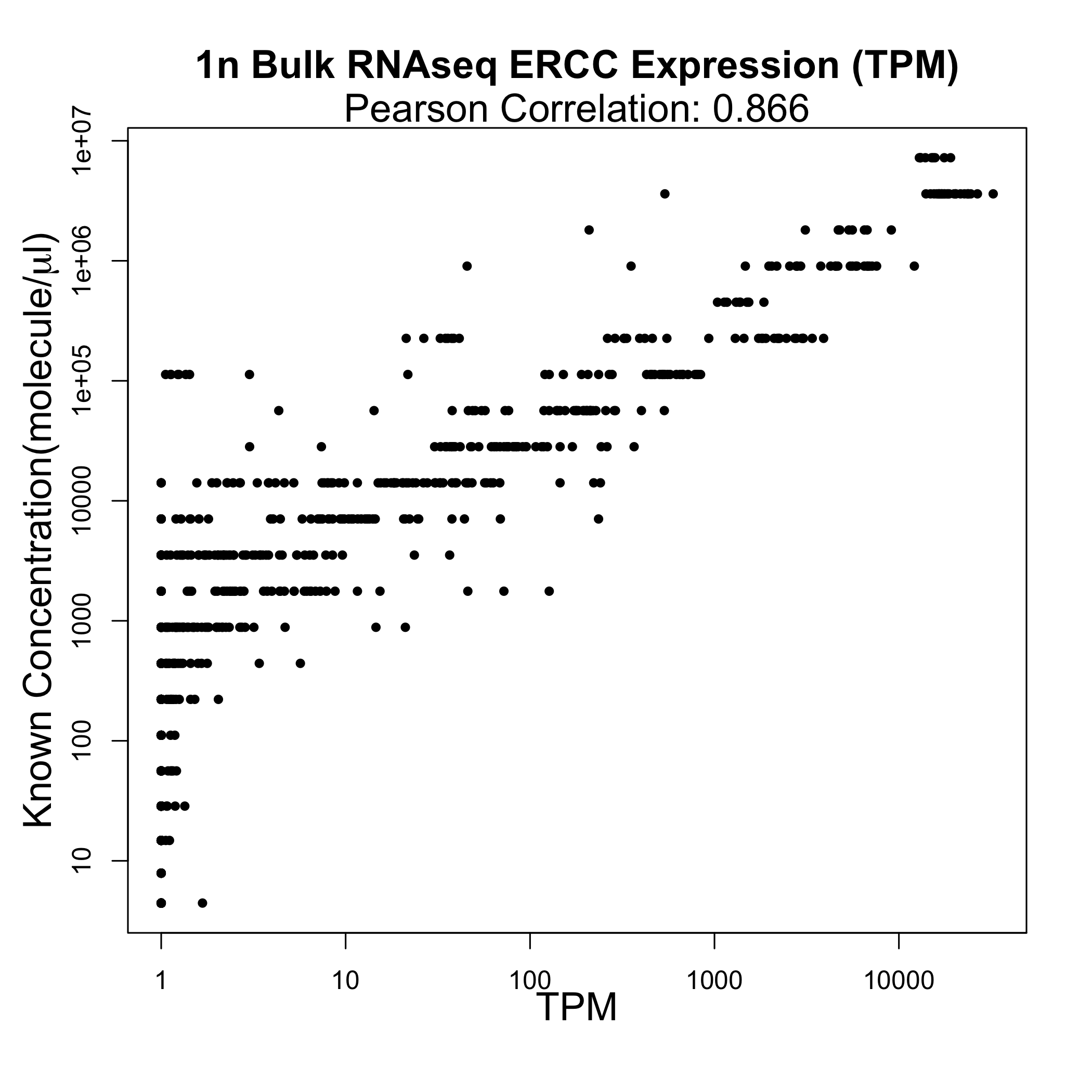

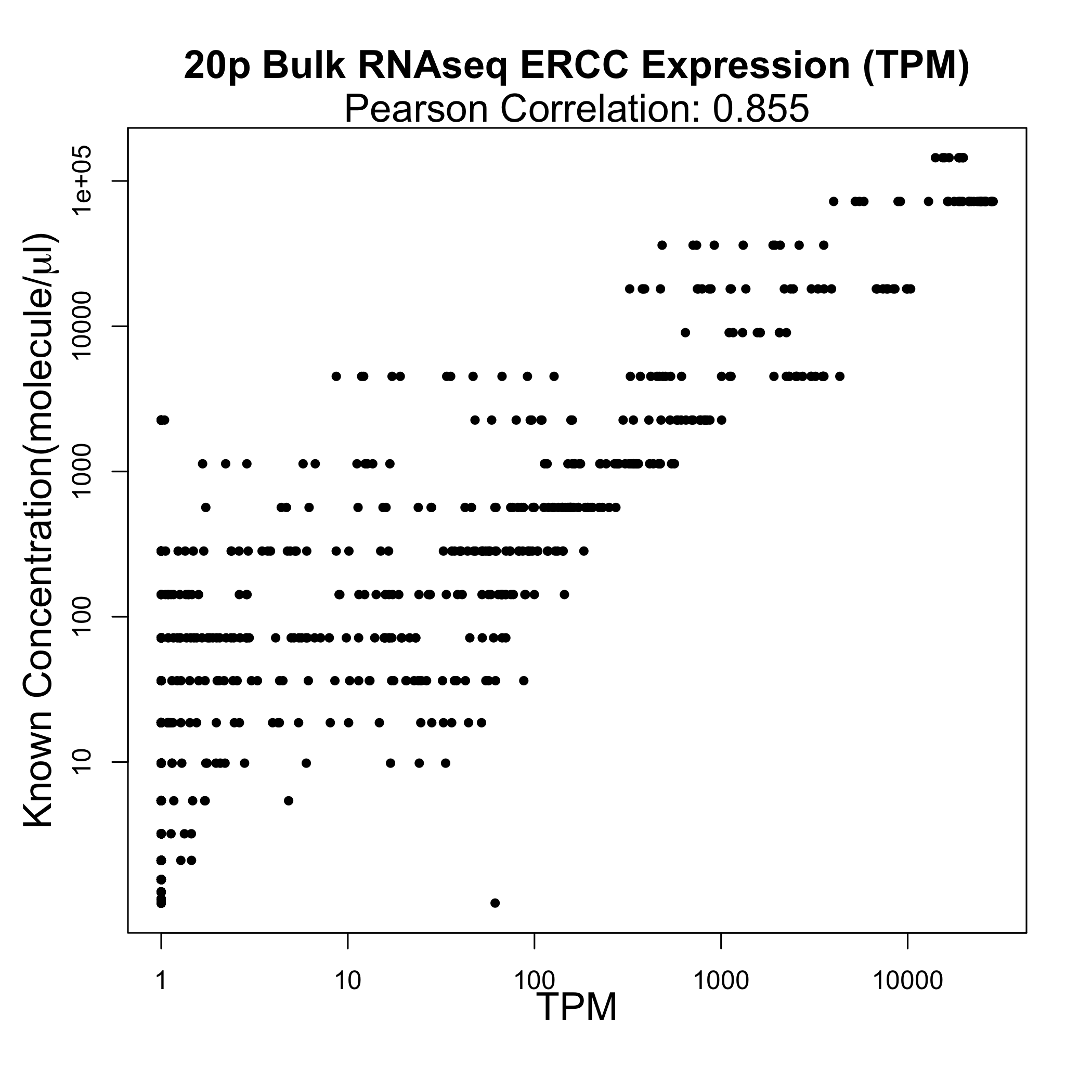

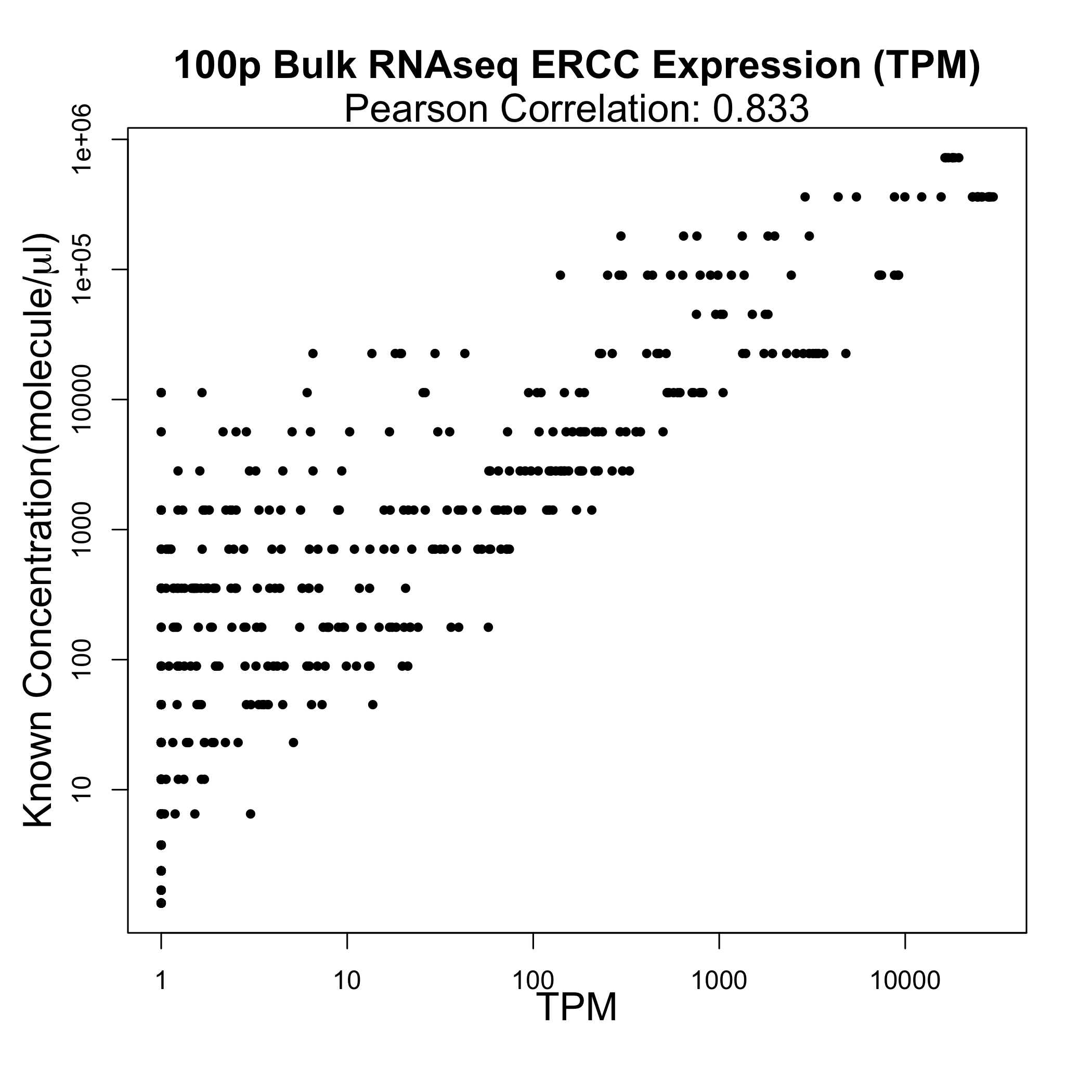

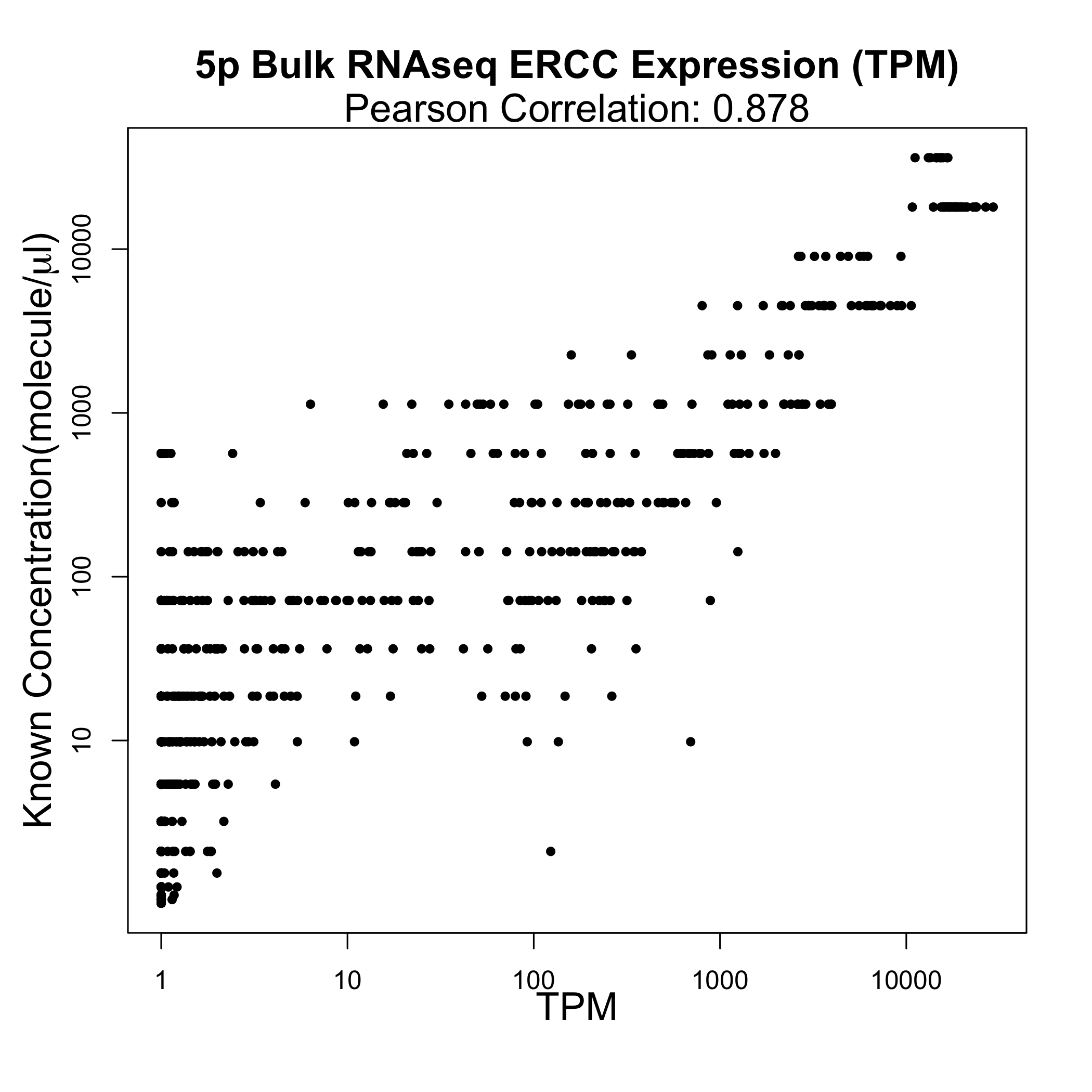

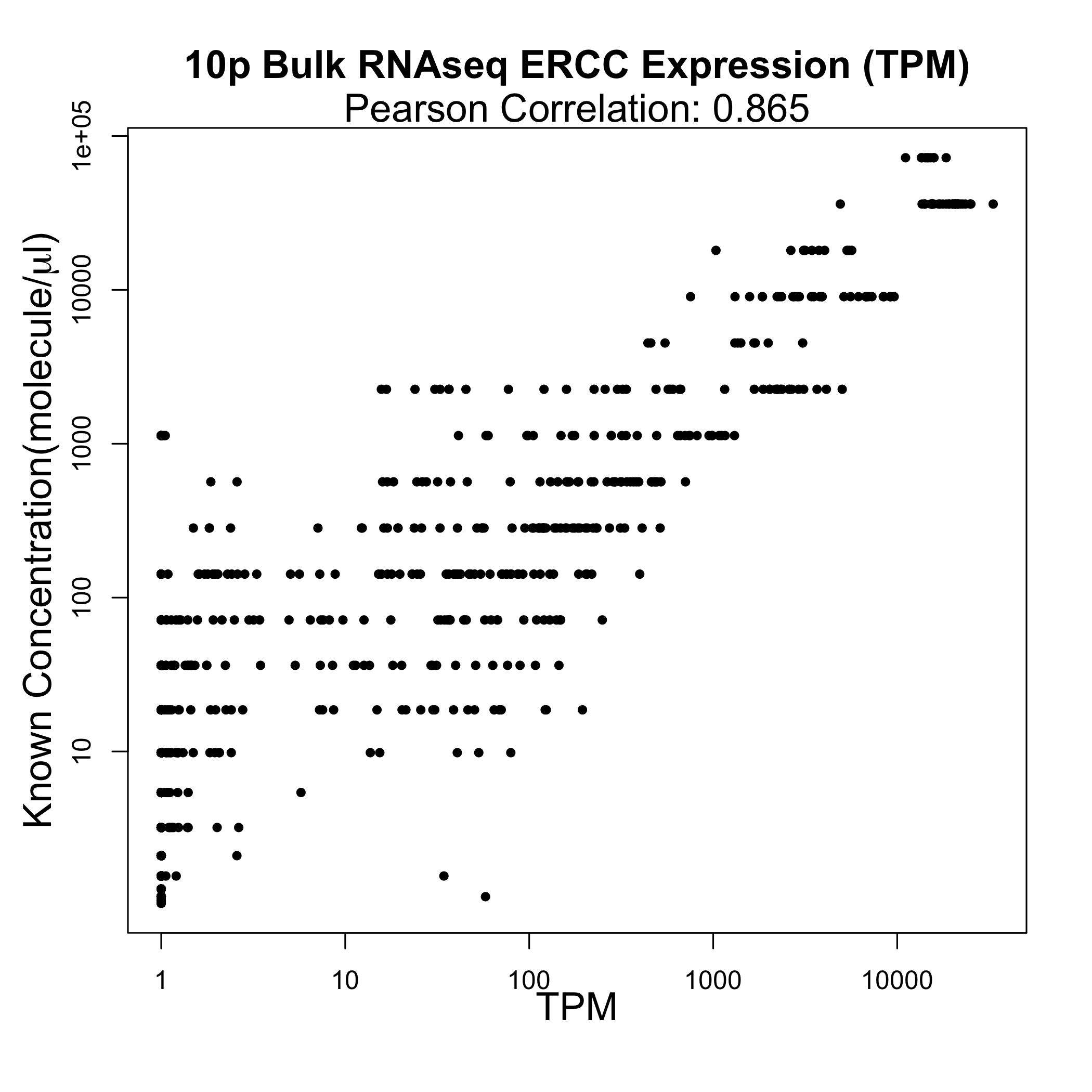


A

B

C

D

E

F


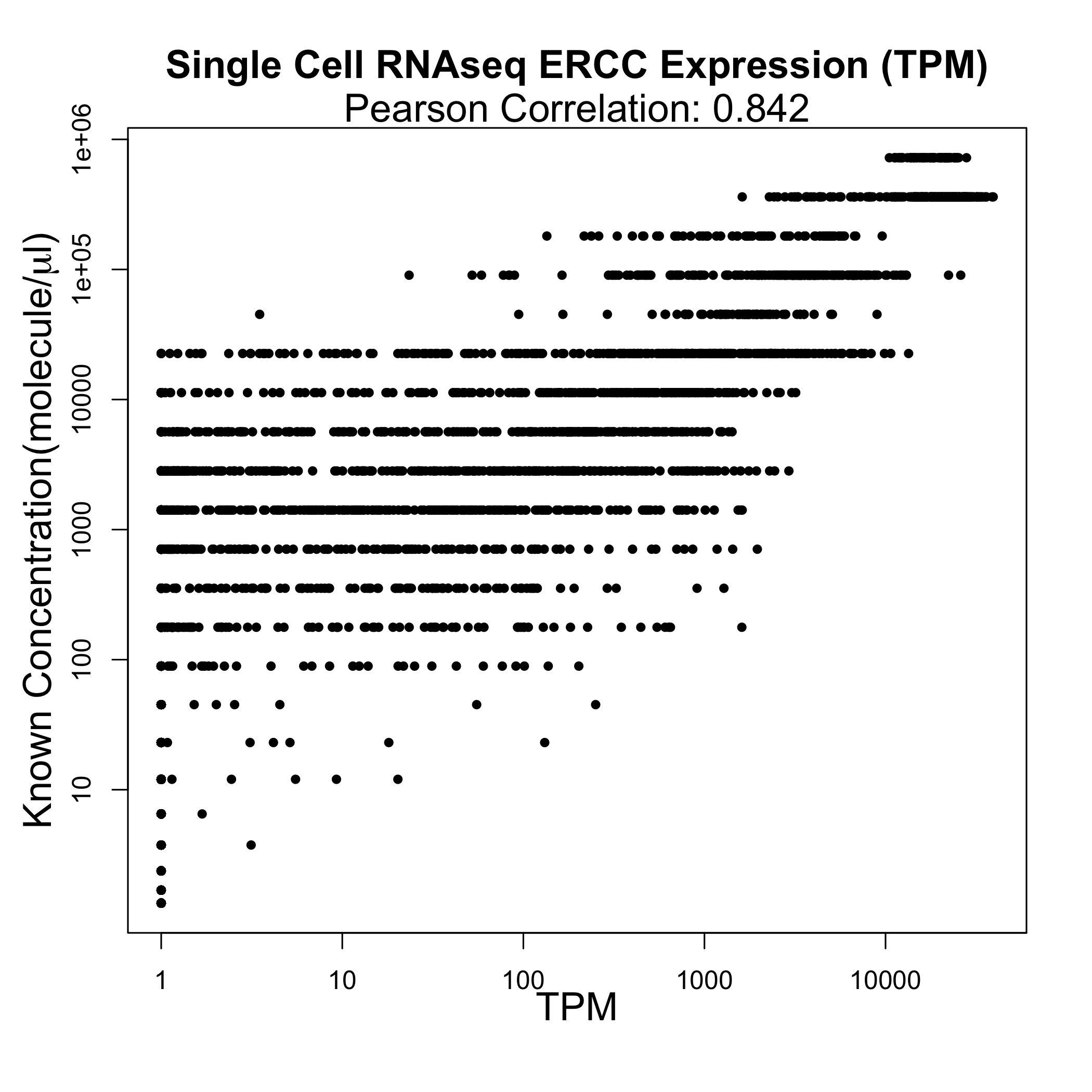


G

**Detection Limit**


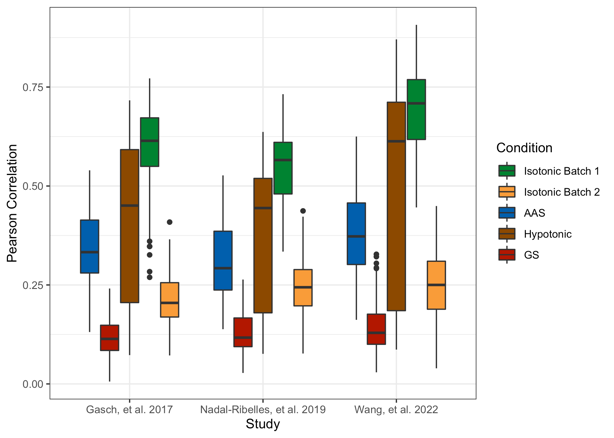


H

I


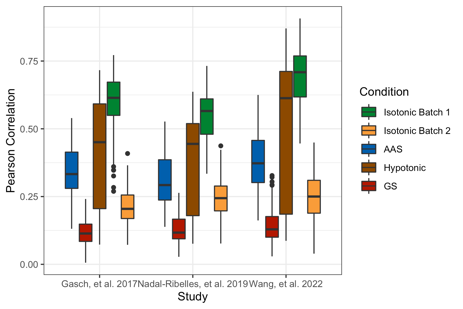


AAS

GS

Spearman Correlation Correlation

A

B


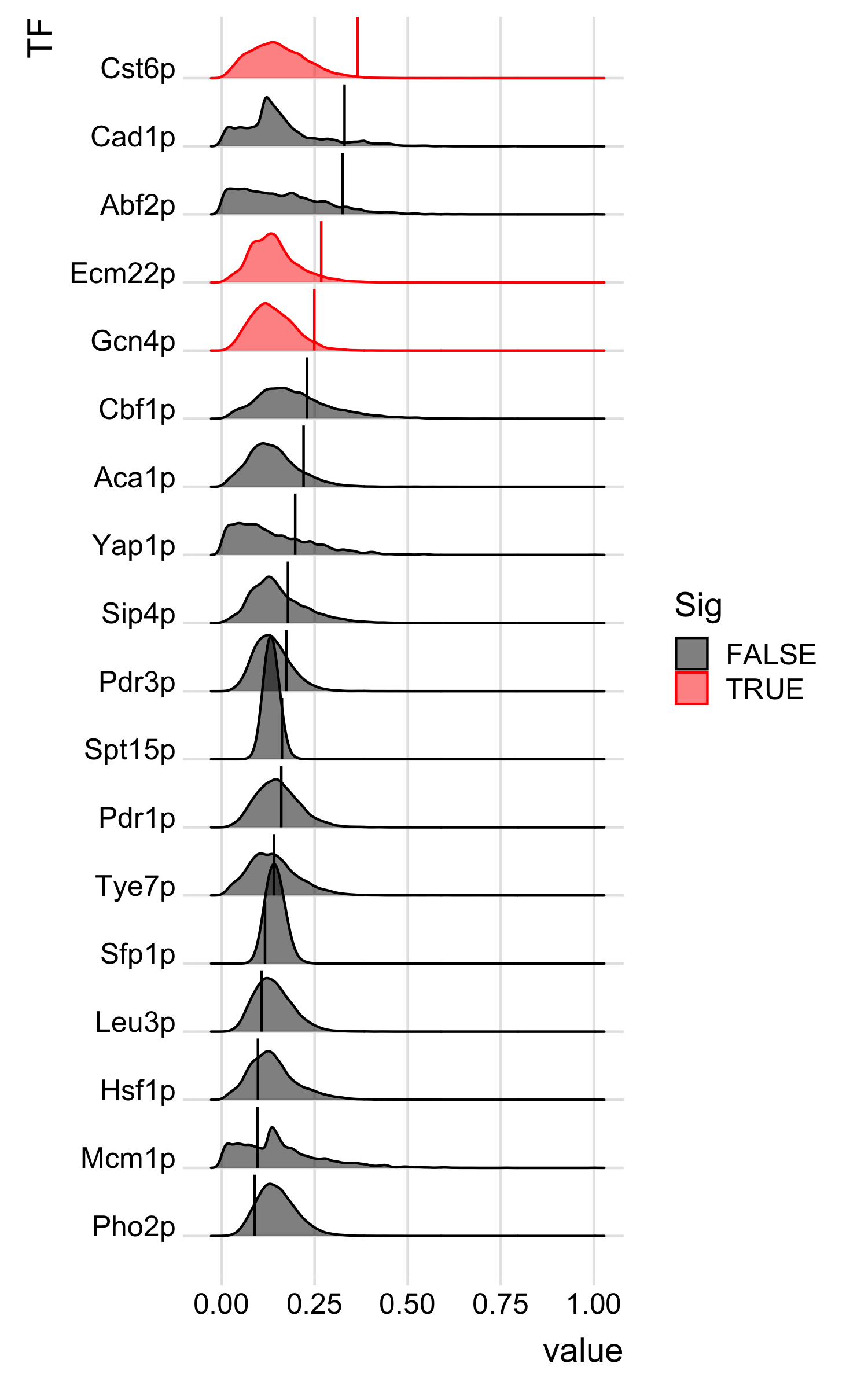

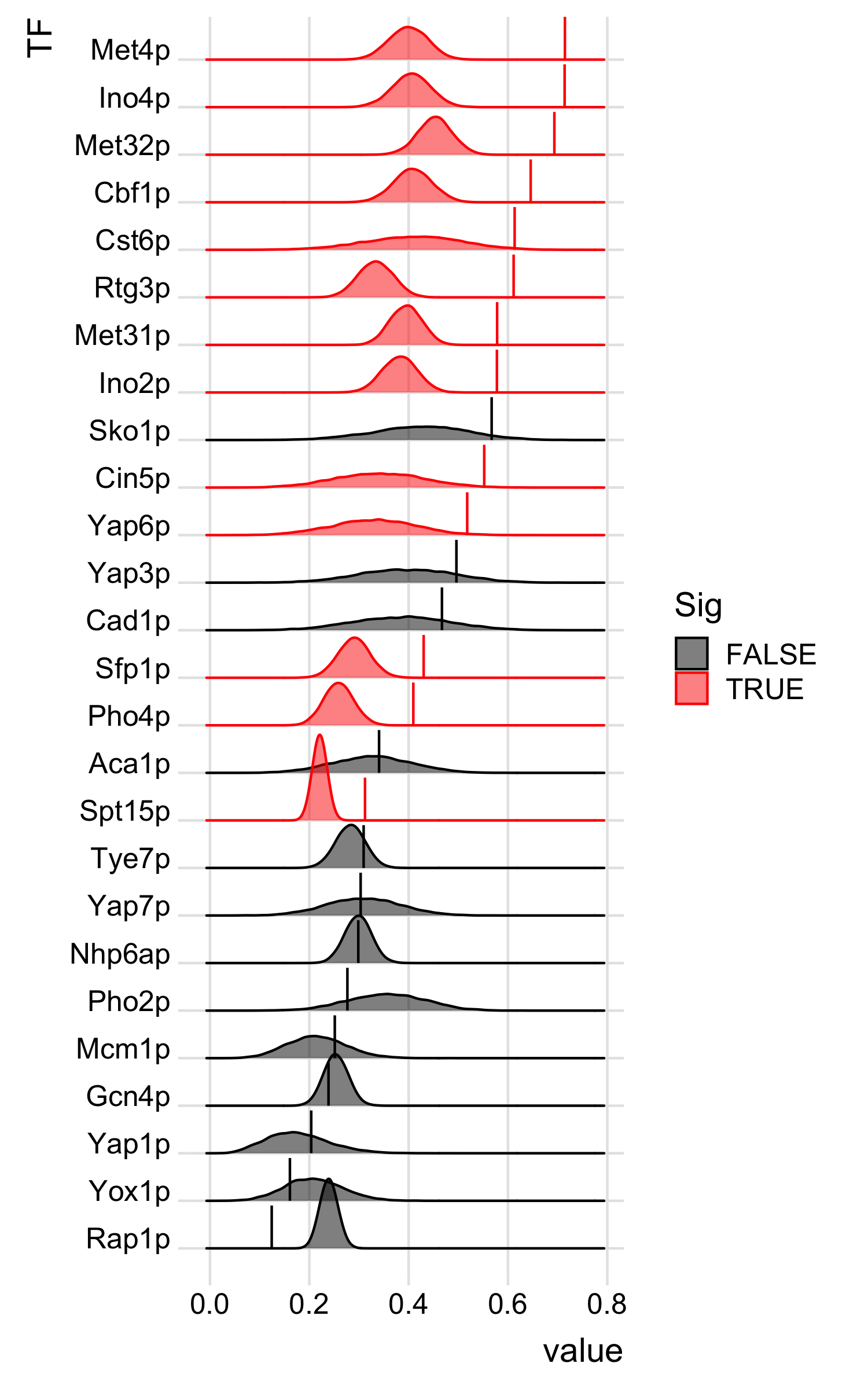


**Supplementary Fig S2. Spearman correlation significance test between predicted target DEGs and TFs. A**. AAS TF mean spearman correlation with predicted target DEGs vs random gene sets of the same size. **B.** GS TF mean spearman correlation with predicted target DEGs vs random gene sets of the same size. Each ridge-plot shows the distribution of the mean spearman correlation between each TF and randomly sampled gene sets. The vertical bar is the mean spearman correlation between each TF and its predicted DEG targets. The distribution and vertical bars are colored red if the mean spearman correlation between the TF and predicted DEG targets is significantly (alpha = 0.05) greater than expected.


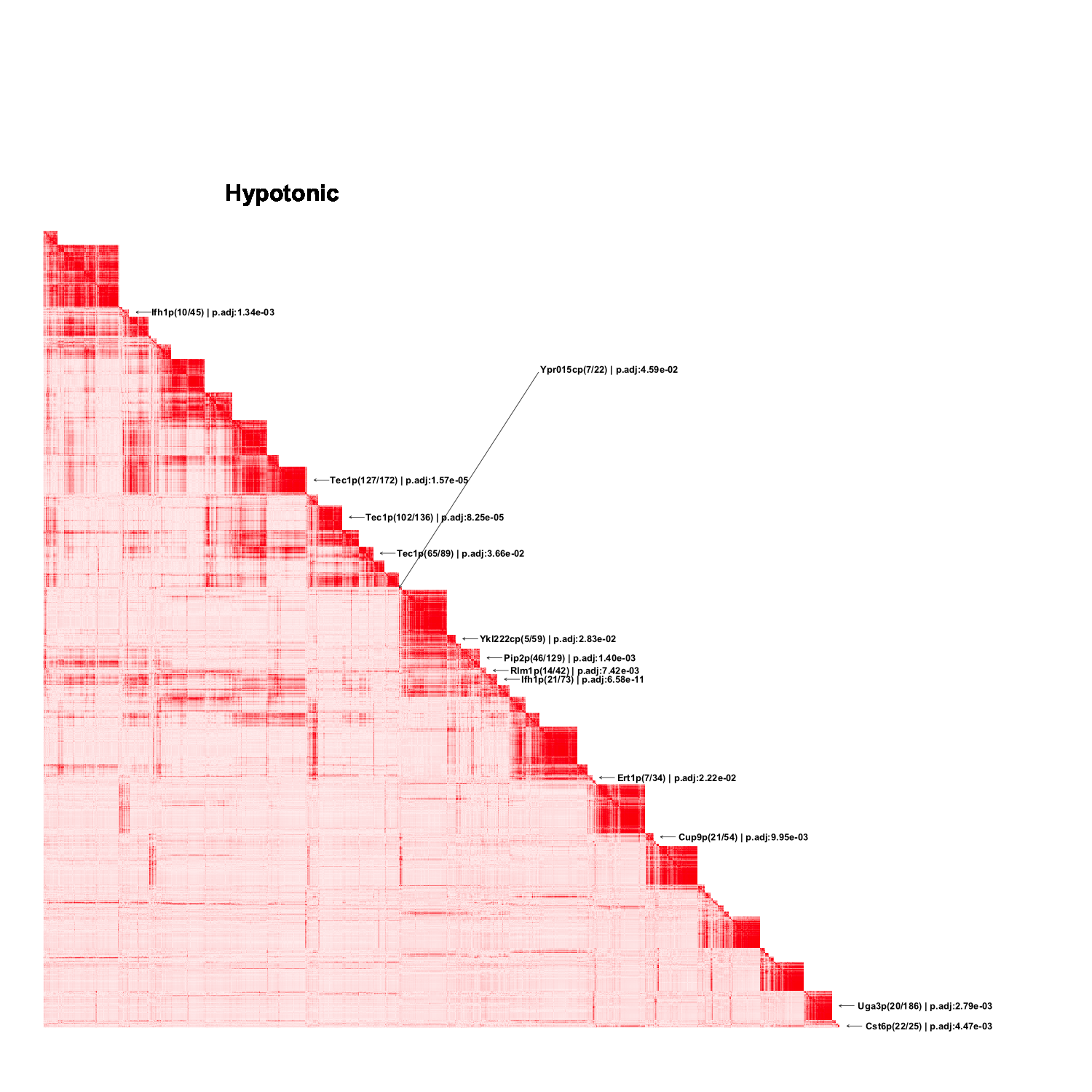

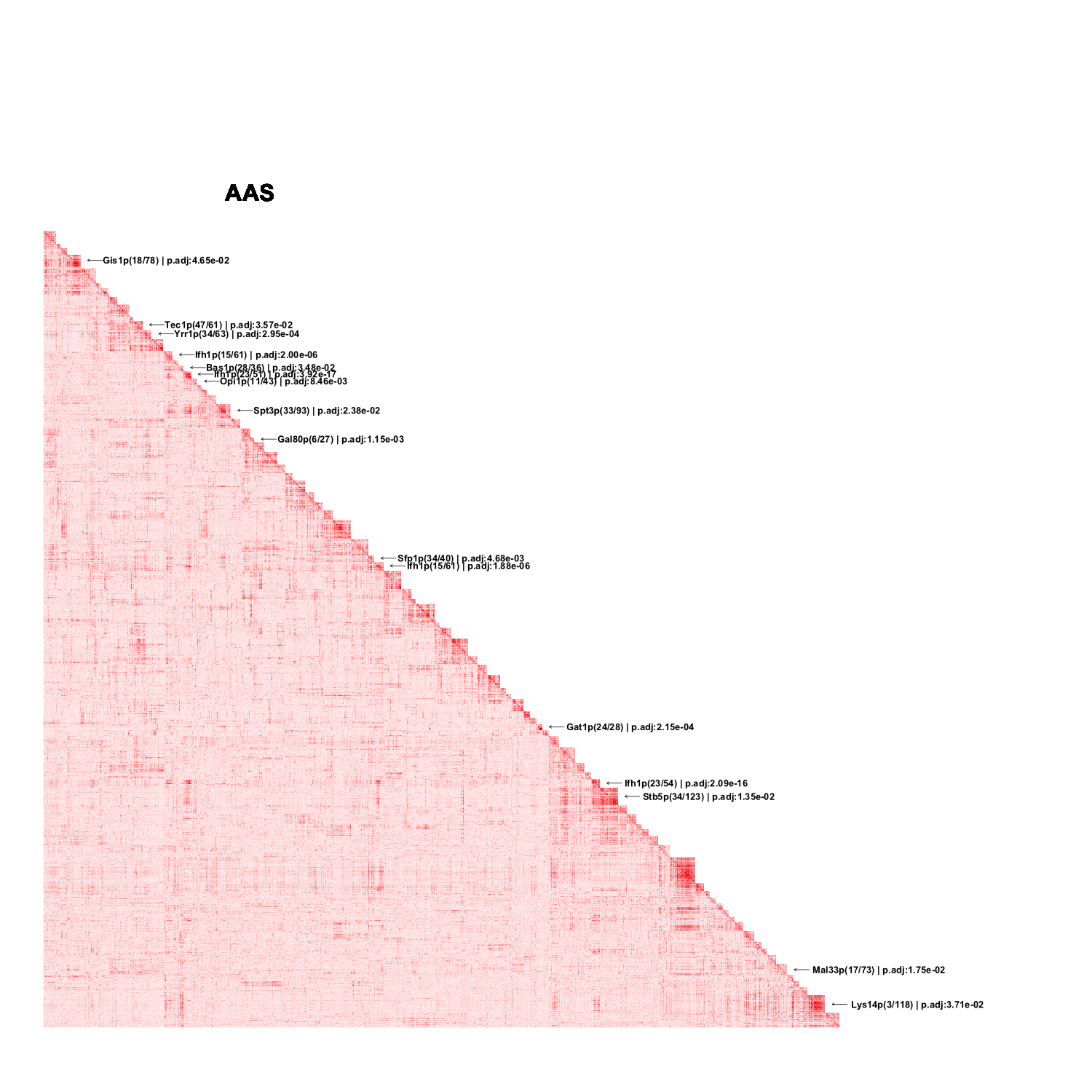


A

B


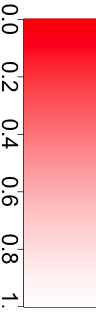


**PCC Distance**


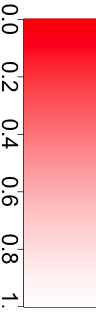


**PCC Distance**


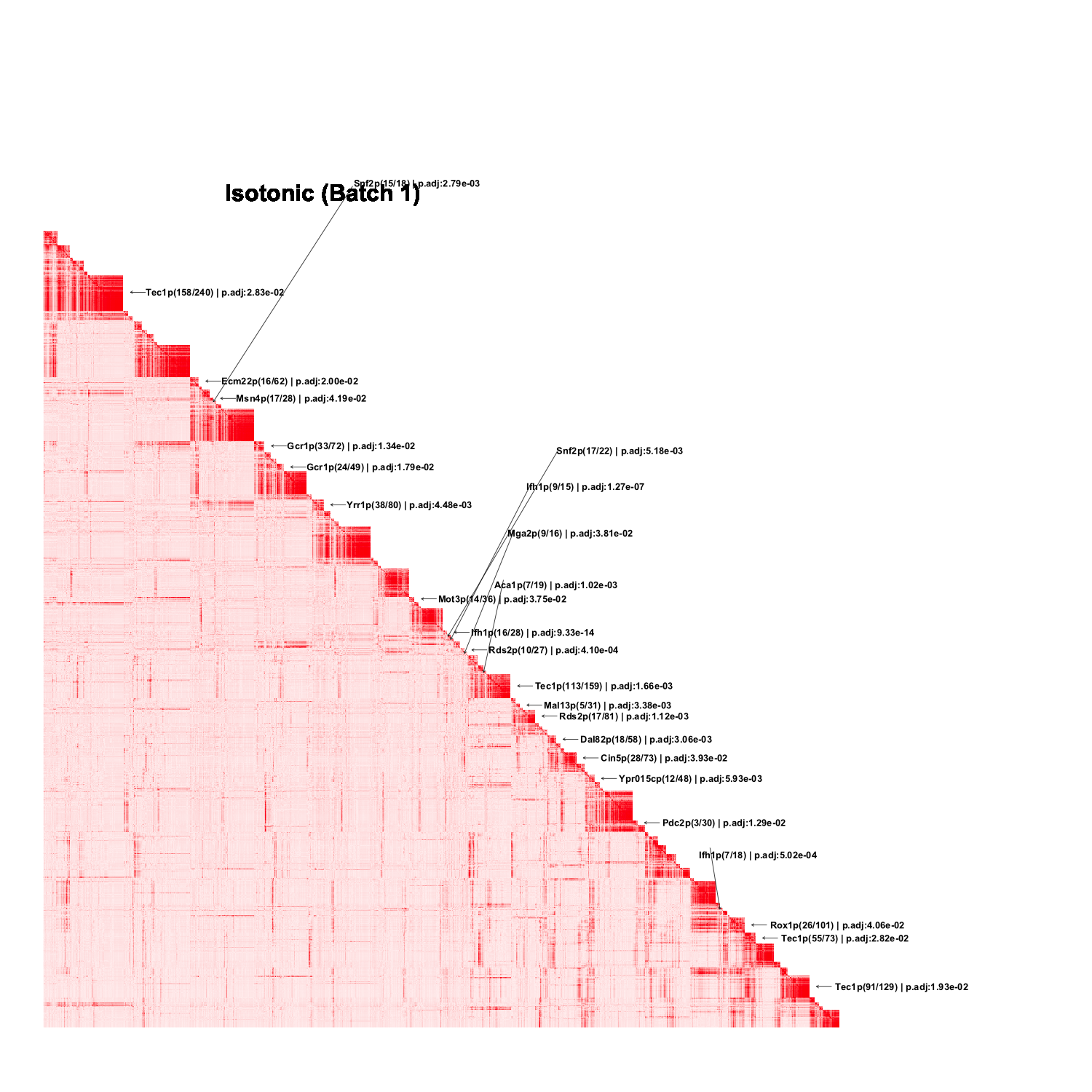


C


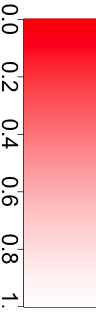


**PCC Distance**

**Supplementary Fig S3.** **Hierarchical clustering of genes under each condition and enrichment of transcription factors for each cluster.** A. AAS. B. Hypotonic. C. Isotonic (Batch 1). Name of the most enriched TF is shown next to cluster if its p.adjust < 0.05. Next to the TF name, the following is shown: (#Genes targets of TF found in cluster)/(#Genes in Yeastract Database found in cluster) | BH adjusted p-value.

**References:**

1. Svensson V, Natarajan KN, Ly LH, Miragaia RJ, Labalette C, Macaulay IC, et al. Power analysis of single-cell RNA-sequencing experiments. Nat Methods. 2017;14(4):381-7.

2. Gasch AP, Yu FB, Hose J, Escalante LE, Place M, Bacher R, et al. Single-cell RNA sequencing reveals intrinsic and extrinsic regulatory heterogeneity in yeast responding to stress. PLoS Biol. 2017;15(12):e2004050.

3. Nadal-Ribelles M, Islam S, Wei W, Latorre P, Nguyen M, de Nadal E, et al. Sensitive high-throughput single-cell RNA-seq reveals within-clonal transcript correlations in yeast populations. Nat Microbiol. 2019;4(4):683-92.

4. Wang J, Sang Y, Jin S, Wang X, Azad GK, McCormick MA, et al. Single-cell RNA-seq reveals early heterogeneity during aging in yeast. Aging Cell. 2022;21(11):e13712.
